# Interplay of halogen bonding and solvation in protein–ligand binding

**DOI:** 10.1016/j.isci.2024.109636

**Published:** 2024-03-29

**Authors:** Maria Luisa Verteramo, Majda Misini Ignjatović, Rohit Kumar, Sven Wernersson, Vilhelm Ekberg, Johan Wallerstein, Göran Carlström, Veronika Chadimová, Hakon Leffler, Fredrik Zetterberg, Derek T. Logan, Ulf Ryde, Mikael Akke, Ulf J. Nilsson

**Affiliations:** 1 Department of Chemistry, Lund University, Lund, Sweden; 2 Microbiology, Immunology, and Glycobiology, Department of Experimental Medicine, Lund University, Lund, Sweden; 3 Galecto Biotech AB, Gothenburg, Sweden

**Keywords:** Thermodynamics, Computational chemistry, Quantum chemical calculations, Nuclear magnetic resonance, Biochemistry

## Abstract

Halogen bonding is increasingly utilized in efforts to achieve high affinity and selectivity of molecules designed to bind proteins, making it paramount to understand the relationship between structure, dynamics, and thermodynamic driving forces. We present a detailed analysis addressing this problem using a series of protein–ligand complexes involving single halogen substitutions — F, Cl, Br, and I — and nearly identical structures. Isothermal titration calorimetry reveals an increasingly favorable binding enthalpy from F to I that correlates with the halogen size and σ-hole electropositive character, but is partially counteracted by unfavorable entropy, which is constant from F to Cl and Br, but worse for I. Consequently, the binding free energy is roughly equal for Cl, Br, and I. QM and solvation-free-energy calculations reflect an intricate balance between halogen bonding, hydrogen bonds, and solvation. These advances have the potential to aid future drug design initiatives involving halogenated compounds.

## Highlights

• Study on halogenated ligand-protein complexes advances understanding of X-bonds
• Enthalpies correlate with halogen bond strength
• Halogen–dependent solvation ΔG of free ligands influences affinity
• Halogen–dependent solvation ΔG of ligand-protein complexes influences affinity
• Cl and Br offer the optimal balance of halogen bond strength and solvation-free energy

## Graphical abstract

**Figure:**
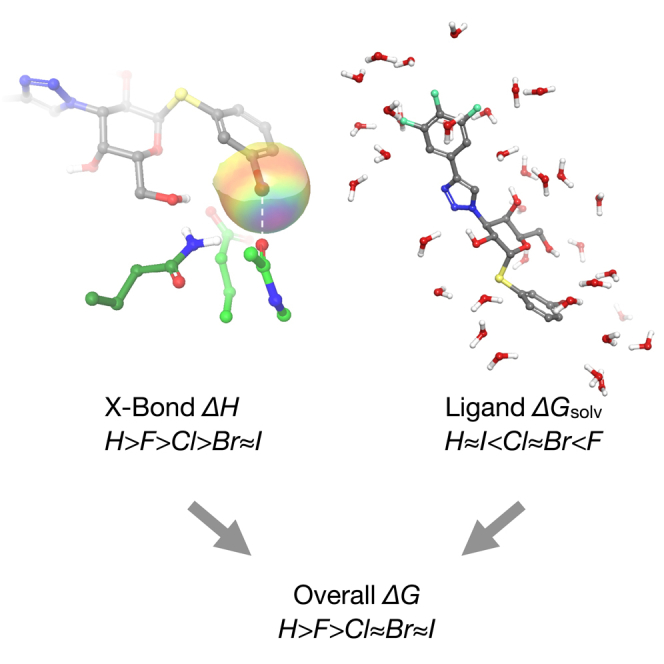

## Introduction

Design and discovery of small molecules with high affinity and selectivity for a given protein is a multi-facetted exercise of optimizing molecular geometry, intermolecular interactions, and chemical-physical properties toward electronic and shape complementary to the protein binding site, while keeping in mind the effects of solvation and conformational entropy. Protein–molecule interactions occur via a multitude of intermolecular forces, such as hydrogen bonds, dispersion forces, dipole interactions, and electrostatic interactions. The description of interaction modes has diversified over the years and increasing interest has been directed to less common interactions of varying strength, such as cation–π interactions, sulfur–π interactions, halogen bonds, chalcogen bonds, and orthogonal multipolar interactions.^1^ Among these interactions, halogen bonds (XB) have emerged as a fruitful strategy due to their potential to enhance both affinity and selectivity for a target protein in combination with halogen-induced increased hydrophobicity, which favorably influences ADME-PK properties.^2^^,^^3^ Analogously to the hydrogen bond, an XB refers to a non-covalent directional bond between a donor halogen atom (X = Cl, Br, or I) and an acceptor Lewis base.^4^ The halogen atom is generally characterized by an $s^{2}p_{x}^{2}p_{y}^{2}p_{z}^{1}$ configuration, where $p_{z}^{1}$ engagement in a covalent s-bond, typically to a carbon of an arene, depletes electron density on the face of the halogen opposite to the s-bond. The resulting electron deficiency is commonly termed the “σ-hole,” which consequently can act as an electron acceptor. Hence, electron donation from a lone pair, typically contributed by a carbonyl oxygen, to the electropositive σ-hole can lead to a favorable n–σ∗ interaction. The σ-hole is circumscribed by a hydrogen bond-accepting electronegative belt, making the halogen amphoteric.^5^^,^^6^^,^^7^^,^^8^^,^^9^ The electropositive character of the σ-hole is proportional to the polarizability of the halogen atom, which increases with size and decreases with the electronegativity, resulting in the order Cl < Br < I, while F does not have a significant σ-hole. Consequently, halogens can form short C–X···O–Y interactions with proteins (where O–Y is a protein carbonyl, hydroxyl, charged carboxylate or phosphate group) in a nearly linear C–X···O arrangement. In addition, the electronegative belt can accept hydrogen bonds from e.g., hydroxyl groups or water molecules and form hydrogen bond-enhanced XB (He-XB). Water molecules involved in He-XB can also form bridges from the halogen to the XB donor atom (e.g., C–X···H–O–H···O–Y).^6^^,^^10^^,^^11^^,^^12^ The formation of He-XB has been found to stabilize a variety of protein–ligand conformations and their importance in biological systems has been analyzed in detail).^8^ Finally, a survey of X-ray crystal structures of biological macromolecules with bound halogenated inhibitors showed that one-third of the analyzed structures had oxygen–halogen distances shorter than the sum of their van der Waals radii, clearly indicating the presence of XB,^13^ which has stimulated interest in understanding and exploiting XB in drug discovery.^14^^,^^15^ Although the XB is comparatively well understood in terms of the σ-hole n–σ∗ interaction, the influence of the halogen atom is less well understood when it comes to *e.g*., water bridges in He-XB, crowding, and possible concurrent effects on protein–ligand complexes.

**Figure 1:**
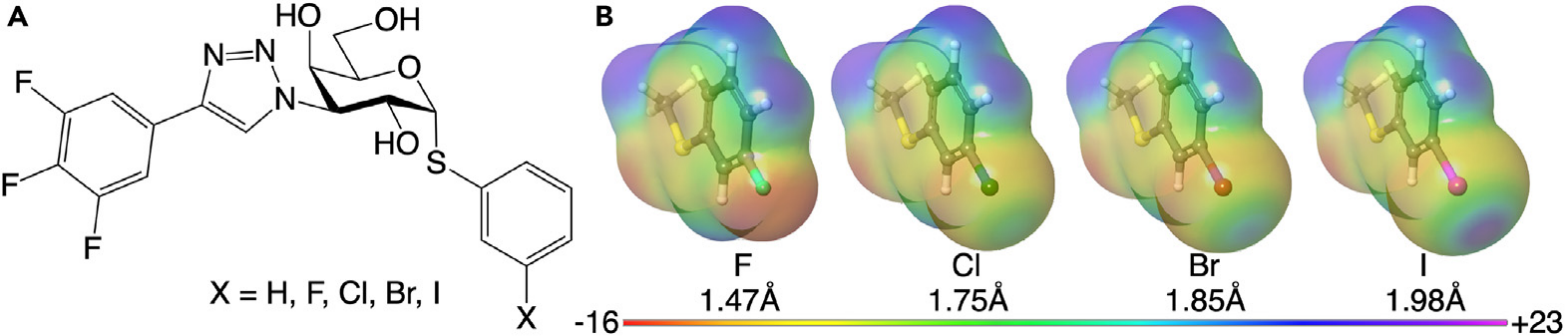
Chemical structures of the ligands **(**A) Structure of the hydrogen and halogen derivatives **H**, **F**, **Cl**, **Br**, and **I**. (B) The electrostatic potential (in kJ/mol/C) mapped on the electron-density surface of the halophenyl aglycon methyl sulfide of the **F**, **Cl**, **Br**, and **I** ligands. The van der Waals radii of the halogen atoms are noted below each surface.

Here we address the question of how the choice of halogen might influence protein–ligand binding mechanisms and thermodynamics, and how the presence of He-XB and surrounding water molecules moderate these characteristics. We address these questions by comparing ligand binding to galectin-3 across a ligand series involving a single change from H, F, Cl, Br, to I. *m*-Halophenyl galactoside ligands targeting galectin-3 were recently demonstrated to form an affinity-enhancing XBs to the backbone carbonyl oxygen of Gly182^16^ and thus serve as an ideal scaffold for an H, F, Cl, Br, and I ligand series (Figure 1). We apply a comprehensive thermodynamic analysis of the variation in binding affinity and relate this to structural differences among the protein–ligand complexes, combining experimental and computational methods; isothermal titration calorimetry (ITC), X-ray crystallography, NMR spectroscopy, quantum mechanical (QM) calculations and molecular dynamics simulations (MD) with grid inhomogeneous solvation theory (GIST) analysis. Our results advance the understanding of how the XB structure, thermodynamics, and solvation are influenced by the choice of halogen atom.

## Results and discussion

### The binding enthalpy is favorable and increases progressively with halogen polarizability and **σ**-hole electropositive character

**Figure 2:**
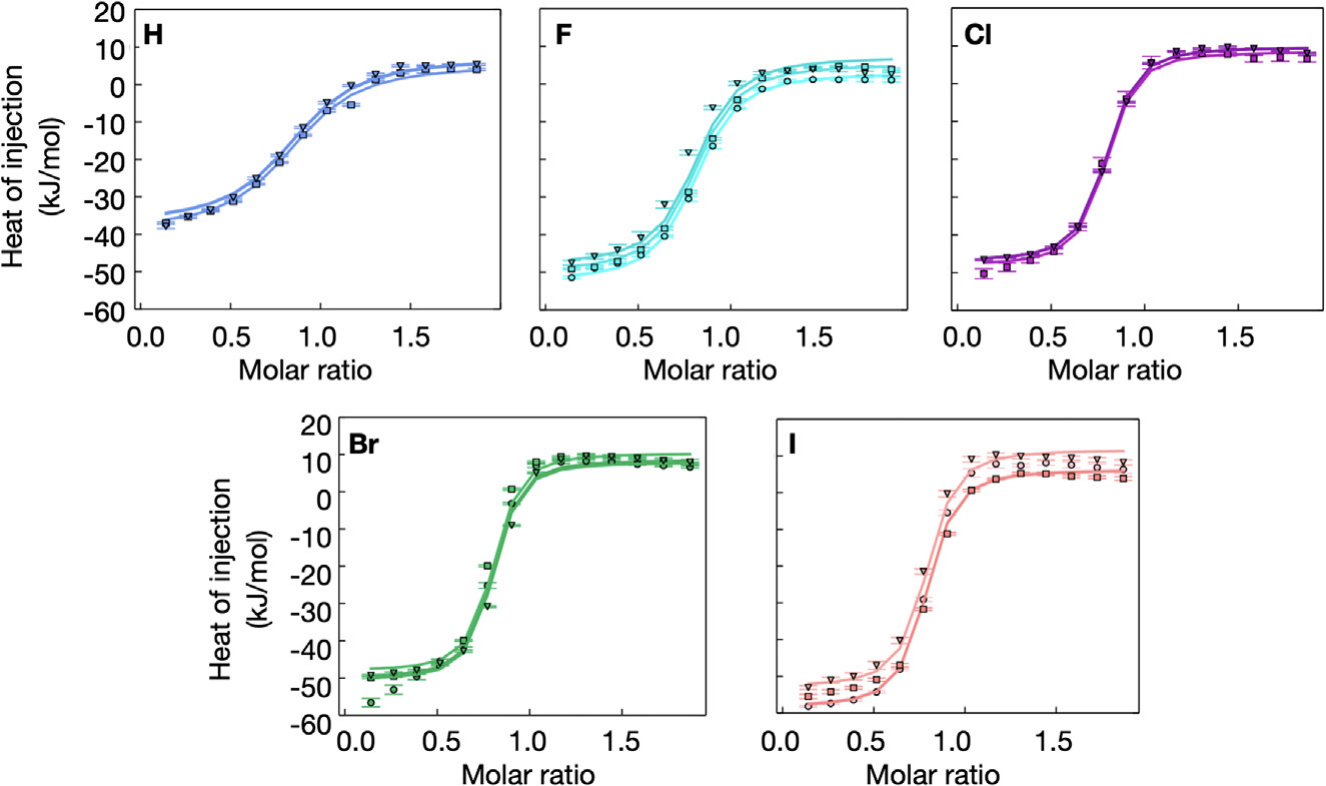
ITC experiments characterizing ligand binding to galectin-3C Triplicate isotherms are shown describing the titration of galectin-3C with ligands **H**, **F**, **Cl**, **Br**, and **I**. The thermodynamic binding parameters result from the global fitting of the three replicate datasets. The graphs are color coded to indicate the ligand: **H** (slate), **F** (cyan), **Cl** (magenta), **Br** (green), and **I** (salmon pink). Errors are estimated from baseline uncertainties.^17^

We used ITC to determine the overall binding thermodynamics for the complexes between galectin-3C and each of the ligands **H**, **F**, **Cl**, **Br**, and **I** (Figure 2). In the case of **Br** and **I**, the data at the end of the titration show a weak trend toward a positive heat of injection that might reflect a secondary process; given its minor contribution to the overall titration curve we did not attempt to model this effect.

All four halogenated ligands **F**, **Cl**, **Br**, and **I** displayed improved affinity over **H** with dissociation constants in the sub-micromolar range (Table 1). The ITC-derived dissociation constants correlate well with those obtained by competitive fluorescence anisotropy experiments with intact galectin-3,^16^ albeit with higher values but unchanged relative affinities, as previously found for other ligand–galectin-3C complexes.^18^^,^^19^^,^^20^ The enthalpy of binding measured by ITC increases progressively from **H** to **I**. The trend within the halogen series follows the increasing halogen polarizability and σ-hole electropositive character, which might be expected to strengthen the XB with the Gly182 backbone carbonyl oxygen observed in the crystal structures (see later in discussion).

**Table 1:** Overall binding thermodynamics from ITC for H, F, Cl, Br, and I

Ligand^a^	*K*_d_ (μM)	Δ*H* (kJ/mol)	–*T*Δ*S* (kJ/mol)	Δ*G* (kJ/mol)
**H**	1.23 ± 0.30	−43.9 ± 2.1	9.8 ± 2.5	−34.1 ± 0.4
**F**	0.48 ± 0.15	−55.3 ± 2.5	18.8 ± 2.5	−36.4 ± 0.8
**Cl**	0.22 ± 0.06	−56.6 ± 1.2	18.4 ± 1.7	−38.3 ± 0.8
**Br**	0.17 ± 0.09	−58.4 ± 3.3	19.4 ± 3.3	−39.0 ± 1.2
**I**	0.22 ± 0.07	−64.5 ± 2.1	26.2 ± 2.5	−38.3 ± 0.8

a ITC results are determined from a global fit to data from three experiments at a temperature of 301 K. *K*_d_ = dissociation constant.

### The binding entropy is unfavorable and varies in discrete steps from H to (F, Cl, Br) and then to I

The free energies of binding do not follow the same trend as the enthalpy, but instead show the highest affinity for the **Br** derivative (Table 1). The entropy decreases in two steps: first from **H** to **F** and then from **Br** to **I**, whereas it stays essentially constant for **F**, **Br**, and **Cl**. The favorable enthalpic contribution observed for the **I** derivative is counteracted by a significantly more unfavorable entropy of binding, such that the net binding free energy is on par with that of **Cl** and **Br** (Table 1). To understand the molecular basis for these results, we determined the crystal structure of each complex, characterized ligand binding using NMR spectroscopy, performed QM calculations of the interaction and solvation energies, and estimated the effects of changes in solvation-free energy using GIST calculations based on MD simulations.

### Structural analysis reveals minute protein structure changes but altered solvation

**Figure 3:**
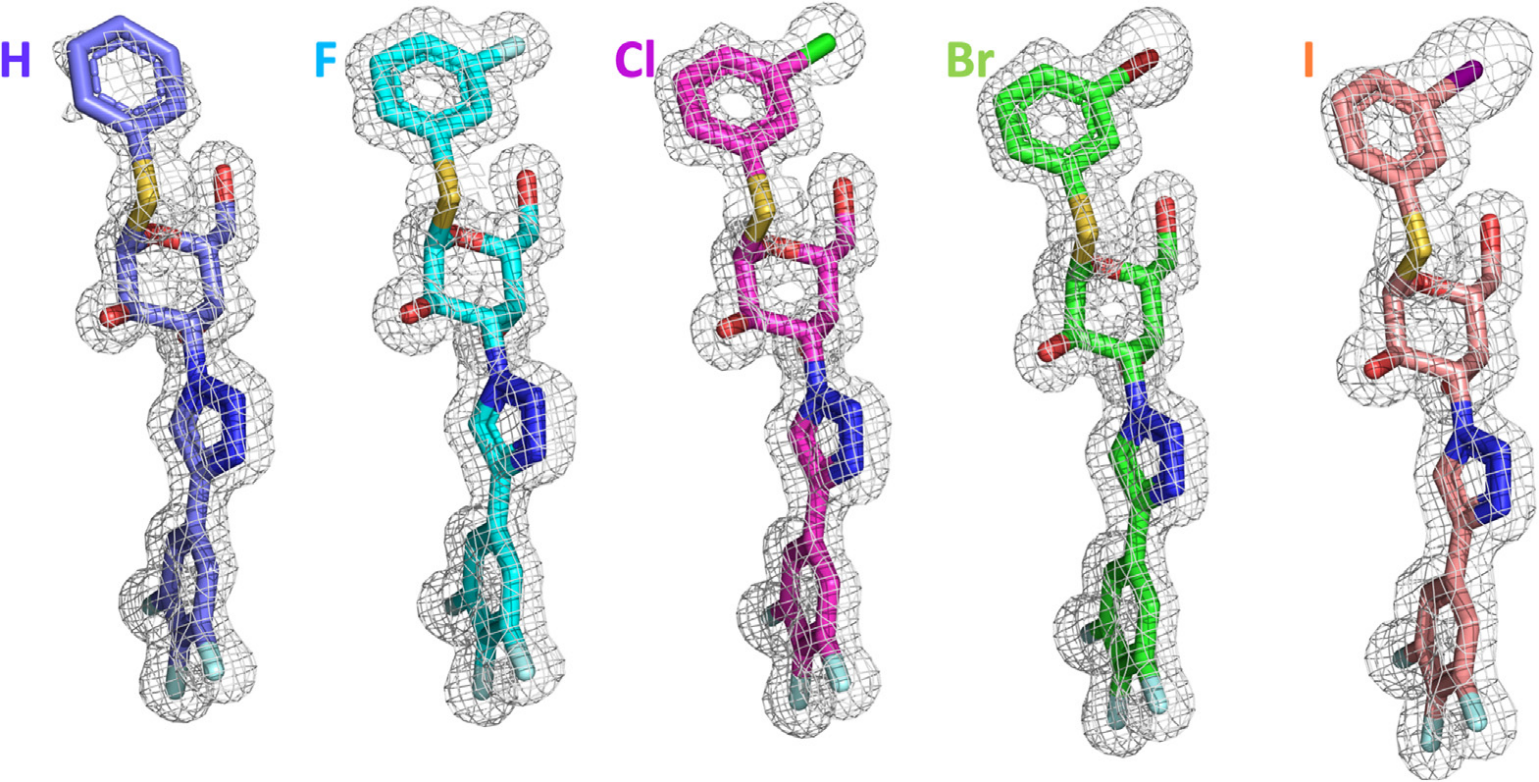
Isomesh view of the ligands depicting their clear electron densities in complex with galectin-3C Ligands are color coded: **H** (slate), **F** (cyan), **Cl** (magenta), **Br** (green), and **I** (salmon pink).

**Figure 4:**
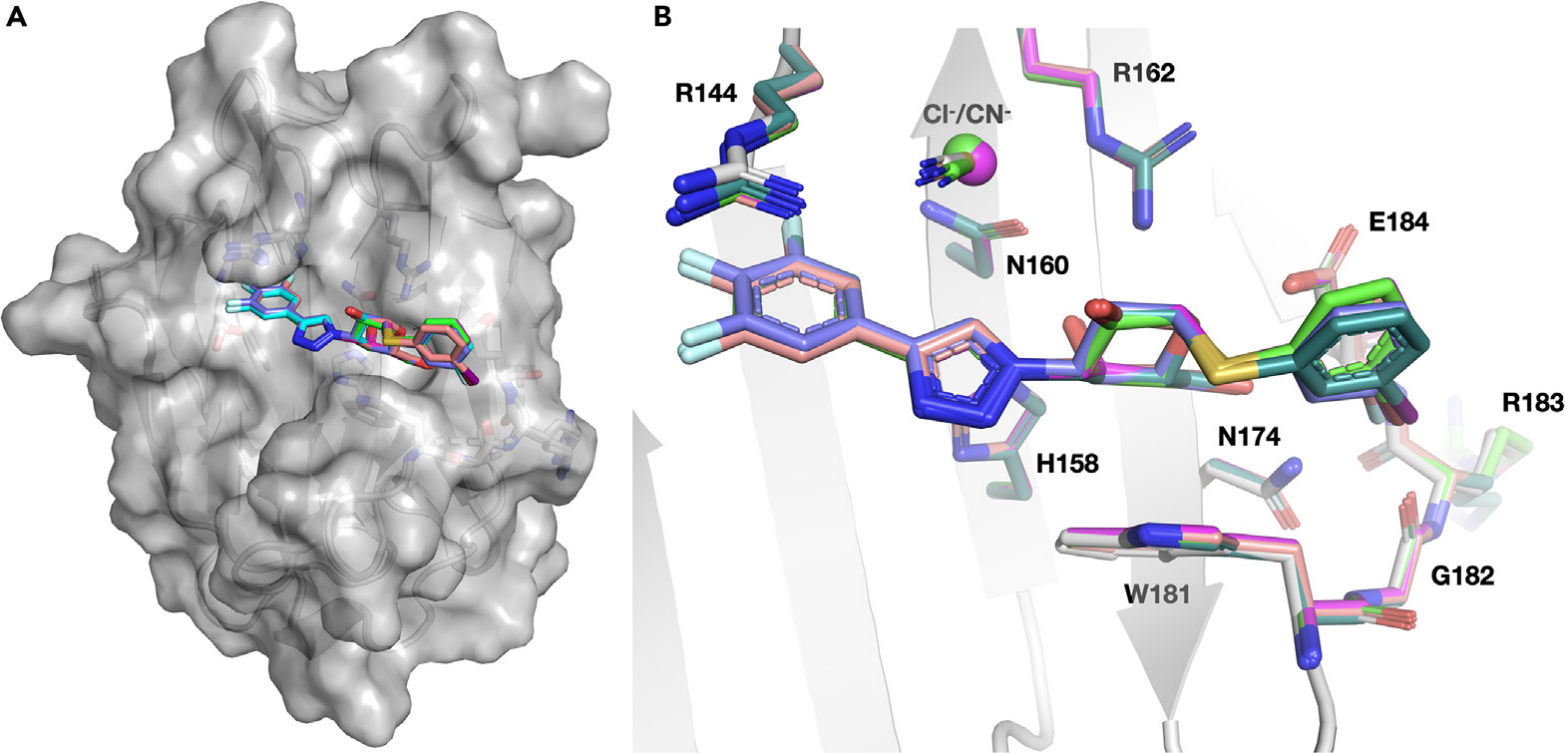
Binding of ligands H, F, Cl, Br, and I to galectin-3C (A) **Surface view** depicting the protein and binding pocket; ligands are shown as sticks. (B) Close-up view of the binding site with key residues and ligands shown. Ligands are color coded: **H** (slate), **F** (cyan), **Cl** (magenta), **Br** (green), and **I** (salmon pink).

We determined crystal structures of each protein–ligand complex in the series **H**, **F**, **Cl**, **Br**, and **I** at high resolution (1.04–1.35 Å, Table S1). All ligands are well defined with clear electron densities (Figure 3). The overall binding pose of all ligands is similar to that previously observed for other α-thiogalactoside-based ligands, with the galactose α-side stacking onto the Trp181 side chain and the ligand C3-trifluorophenyltriazolyl moiety stacking face-to-face with the Arg144 guanidino group (Figure 4).^16^^,^^21^^,^^22^ The phenyl aglycon of the **H** ligand has slightly poorer electron density, most likely due to the lack of XB to Gly182 resulting in increased ligand **H** mobility. This is also reflected in a higher average *B*-factor for the aromatic ring of the **H** ligand (33 Å^2^ compared to 14–18 Å^2^ for the **F**, **Cl,** and **Br** structures, which are of similar resolution). Importantly, all ligands are positioned in galectin-3C with virtually identical binding modes, indicating that the ligand–protein interactions differ only in the XB. Minute differences are observed around key interactions, as described in detail later in discussion.

**Figure 5:**
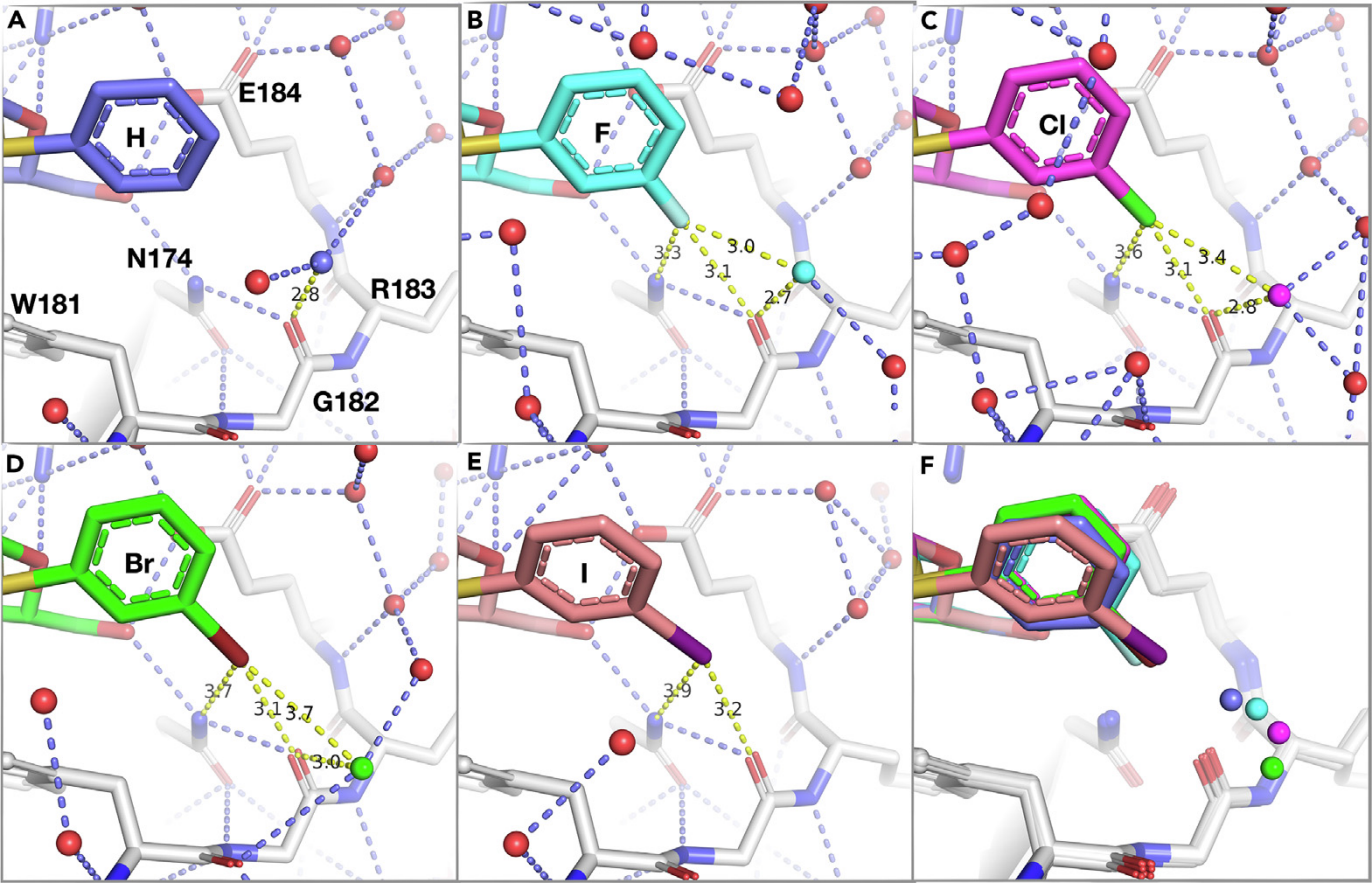
Close-up view of the halophenyl moiety in each ligand–galectin-3C complex Panels (A–E) are labeled with the atom letter (H, F, Cl, Br, or I). Interactions of the halogen, the Gly182 carbonyl oxygen, and the bridging water molecule(s) are indicated with yellow dashed lines and distance in Å. Other hydrogen bonds are indicated with blue dashed lines. (F) Superposition of all 5 complexes depicting the gradual water displacement in the four halogen-containing ligand complexes. Ligands and the variable water molecules near the halogen atom are color-coded: H (slate), F (cyan), Cl (magenta), Br (green), and I (salmon pink). The water molecule is not present in the I complex.

The complex with ligand H has two water molecules close to the ligand phenyl aglycon and the Gly182 carbonyl oxygen (Figure 5A), whereas the complexes with ligands F, Cl and Br have only one water molecule near the ligand halophenyl aglycon. The single water molecule allows for the simultaneous formation of an XB and a water-bridged hydrogen bond with the carbonyl group of Gly182, i.e., an He-XB (Figures 5B–5D). In the complex with ligand I, there is no XB-bridging water molecule present, as the nearest water oxygen atom is 4.4 Å away, indicating that the water–XB interaction for I is less favorable in this case (Figure 5F). The X···O distance to Gly182 is almost identical in all complexes (Figures 5B–5E) with distances of 3.1 Å (Cl and Br) or 3.3 Å (I), which are all less than the corresponding sums of the van der Waals radii (3.27 Å for Cl···O, 3.37 Å for Br···O and 3.50 Å for I···O).^13^ The three ligands F, Cl, and Br, that form a water-bridged He-XB with the Gly182 carbonyl, have the aglycon phenyl placed in nearly identical positions, while the H and I ligands, which lack the water-mediated hydrogen bond, adopt a slightly tilted position of the aglycon phenyl as compared to F, Cl, and Br. Notably, the bridging water molecule moves further away from both the halogen center and the carbonyl oxygen of Gly182 as the size of the halogen atom increases. The side chain amide of Asn174 is arranged underneath the ligand phenyl aglycons to donate hydrogen bonds to the ligand galactose O6, the carbonyl oxygen of Gly182, and the ligand halogen, hence being involved in a He-XB with Gly182. The C–X···H–N angle of ligands H to I and Asn174 decreases from 144 to 125°. Finally, the side chain of Glu184 is positioned face-to-face with the phenyl aglycons, forming an anti-parallel dipole alignment to the C–X bond and accepting hydrogen bonds from the ligand galactose HO6 and the side chains of Arg162 and Arg186.

### NMR chemical shift perturbations correlate with binding enthalpy

**Figure 6:**
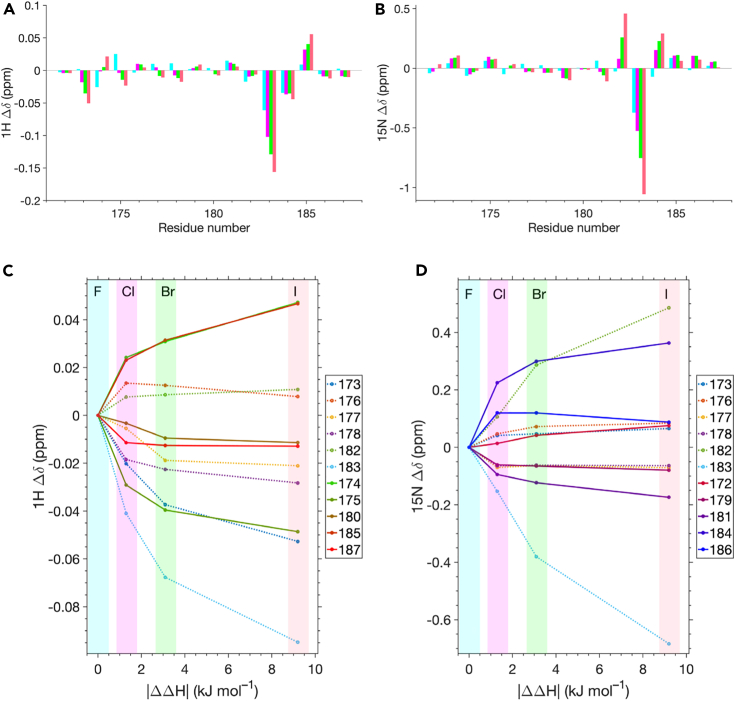
Chemical shift perturbations observed in the four X–galectin-3C complexes (A) Δδ(^1^H) and (B) Δδ(^15^N) plotted versus residue number for residues172–187. (Δδ = δ(**X**-complex) – δ(**H**-complex). The different complexes are indicated by color-coded bars: **F** (cyan), Cl (magenta), **Br** (green), and **I** (salmon pink). (C and D) Chemical shift perturbations plotted versus the difference in enthalpy of binding |ΔΔ*H*| relative to the **F**–galectin-3C complex, (Δδ = δ(**X**-complex) – δ(**F**-complex). The corresponding plots using the H–galectin-3C complex as a reference are shown in Figure S2. For clarity, only residues with Δδ ≥ 0.01 are included.

We monitored the chemical shift differences between the galectin-3C complexes with **H**, **F**, **Cl**, **Br**, and **I** (Figure 6). Figure S1 shows the superimposed ^1^H-^15^N HSQC spectra of galectin-3C in complex with each of the five ligands. The NMR spectra were acquired on samples from the completed ITC experiment. The protein was titrated into the ligand solution in the ITC cell, so that the saturation of the binding reaction monitored by ITC results in free protein present at the end of the titration. Consequently, there is a non-negligible population of apo galectin-3C present in the NMR samples. A separate peak from the apo state was observed for residues that show significant chemical shift perturbations, indicating that ligand binding is in slow exchange on the chemical shift timescale. Hence, the progressive chemical shift perturbations induced by the series of ligands report on the chemical shift of the ligand-bound complex.

Residues with significant chemical shift changes are centered around residues 173–175 and 182–185, in the direct vicinity of the halogen atom (Figures 6A and 6B). The largest chemical shift changes are observed for the backbone amides of Gly182 and Arg183 and the side-chain amide of Asn174 (Figure S1), all of which experience significant perturbations of both the ^1^H and ^15^N chemical shifts. Several other residues show perturbations that are more prominent for one of the two nuclei, but still significant for the other (Figures 6C and 6D). Intriguingly, the chemical shift perturbations in many cases correlate with the enthalpy of binding, indicating that the chemical shifts are sensitive to the strength of the interaction energy between the halogen and the protein atoms (Figures 6C and 6D). This is of particular interest since the crystal structures show that the 5 different complexes have essentially identical structure, with only minute changes around the halogen atom, giving few clues to what interactions drive the difference in binding enthalpy among the complexes. In the following, we attempt to interpret in a qualitative manner the progressive chemical shift changes shown in Figure 6.

The chemical shift arises because of nuclear shielding, induced by surrounding electrons, from the external, static magnetic field. Hence, the chemical shift reflects the electronic structure around the nucleus. Polarizing electric fields that draw electrons away from the atom in question act to deshield the nucleus, leading to an increase in chemical shift, and vice versa. Chemical shift changes induced by XB have been calculated for model compounds using density functional theory, showing that the ^15^N chemical shift of nitrogen atoms in pyridine involved in C–X···N XB decreases linearly with the energy of the XB as it strengthens.^23^ In the complexes studied here, the XB acceptor is the carbonyl of Gly182, so a direct comparison with previous results is not possible due to the difference in acceptor chemistry. However, we expect the general phenomenon that chemical shifts depend linearly on the interaction energy to play a role also in our present case. Furthermore, both ^1^H and ^15^N chemical shifts are known to respond to hydrogen bonding, showing a linear increase in shift with decreasing hydrogen bond length.^24^^,^^25^ For ^15^N, this effect is greater when the preceding carbonyl is involved in hydrogen bonding compared to the case where the HN group itself is hydrogen bonded.^25^ At this level of interpretation, it thus appears that the chemical shift changes in Figure 6 reveal how the interactions in the galectin complexes (considering both the intra-protein interactions, e.g., hydrogen bonds, and the protein–ligand interactions) vary when the halogen atom is substituted from F to Cl, Br, and I.

The side-chain amide of Asn174 shows large positive Δδ(^1^H) and Δδ(^15^N) values compared to the apo state, which increase progressively as the size of the halogen atom increases, indicating progressive deshielding (Figure S1). The crystal structures show that the Asn174 side-chain amide is in contact with the halogen atom in all complexes. This interaction can be described as a hydrogen bond involving as donor the NH_2_ group of Asn174 and as acceptor the electron-rich belt of the halogen atom.^26^^,^^27^ The progressive increase in the ^1^H and ^15^N shifts with increasing size of the halogen atom indicates increased strength of the hydrogen bond. Notably, the two peaks from the H_2_N group are quite weak and only observable for the complexes with **Cl**, **Br**, and **I**, suggesting that the peaks are broadened beyond detection in the case of **H** and **F**. Potential explanations, such as fast amide proton exchange with the solvent water or rapid rotation around the C–N bond, do not appear likely, because the exchange rates of these types of processes are typically much too slow, especially in cases where the NH_2_ group is engaged in a hydrogen bond.^28^^,^^29^ While there is no indication in the crystal structures that the side chain populates alternative conformations, it is still possible that the NH_2_ group undergoes conformational exchange. The progressively decreasing peak broadening in the spectra of **Cl**, **Br**, and **I** suggests that the exchange process is slower in these complexes, further bolstering the concept that the hydrogen bond strength increases with halogen size.

A negative trend in both Δδ(^1^H) and Δδ(^15^N) of the Arg183 backbone amide with increasing halogen size (Figure 6) indicates a progressively stronger shielding of its amide ^1^H and ^15^N, which suggests increased electron density at the H–N moiety with increasing halogen size. The backbone HN of Arg183 is oriented toward the solvent and points away from the ligand. Thus, the chemical shift changes of Arg183 HN likely result from indirect effects due to changes in the polarization of the Gly182 carbonyl oxygen. It might be expected that the Gly182 carbonyl oxygen is donating electron density to the halogen s hole, leading to a “pull” of electron density through the 182–183 peptide bond and deshielding of the Arg183 amide. However, this expectation is opposite to the experimental result. Instead, we speculate that Δδ might be dominated by the increase in distance between the Gly182 carbonyl oxygen and the water molecule near the halogen atom that possibly forms a He-XB, (Figures 5B–5D), which is likely to decrease the polarization of the 182–183 peptide bond and thus lead to increased shielding of the Arg183 amide. Density functional theory calculations of chemical shifts, based on the crystal structures, should aid in interpreting the results, but are outside the scope of the present study.

Several additional residues show smaller chemical shift perturbations with increasing halogen size, which also correlate with the enthalpy of binding (Figure 6). Residues 173–175, 177–179, and 185 all have |Δδ(^1^H)| ≥ 0.02 ppm and 181, 182, and 184 have |Δδ(^15^N)| ≥ 0.125 ppm. Some of these changes can potentially be explained by minor changes in hydrogen-bonding distances across the b-sheet situated underneath the ligand. The backbone amides of Asn174 and Glu185 apparently form shorter hydrogen bonds with increasing halogen size, as revealed by progressively increased ^1^H chemical shifts, while Cys173 and Thr175 show the opposite behavior. Notably, Asn174 and Glu185 form hydrogen bonds in line across the sheet, with Glu185 HN and Asn174 HN hydrogen bonding to Cys173 CO and His158 CO, respectively. A tentative interpretation is that these two hydrogen bonds strengthen progressively as the enthalpy of ligand binding increases, whereas the interactions of the residues on either side of this line appear to become weaker. In fact, Thr175 HN does not form a hydrogen bond, because of a kink in the b-strand at Gly182, but still show signs of decreased polarization. In contrast, Gly182 HN hydrogen bonds to Thr175 CO and this interaction also appears to correlate positively with the enthalpy of binding. Thus, among the hydrogen bonds that attach the outermost strand to the rest of the b-sheet, the two in the direct vicinity of the binding site, viz. Gly182–Thr175 and Glu185–Cys173, apparently become stronger with increasing binding enthalpy. By contrast, the shift changes observed for Trp181, Δδ(^15^N), and Leu177, both Δδ(^1^H) and Δδ(^15^N), might sense a progressively increased hydrogen bond length between Leu177 HN and Asn180 CO and across the b-hairpin. Glu184 N shows an increase in chemical shift, but this HN group is not directed directly toward the binding site and does not form any hydrogen bonds with other protein residues, and neither does the Arg183 carbonyl oxygen. However, the backbone HN of Glu184 might respond to adjustments in hydrogen bonding to another conserved water molecule than the one bridging to Gly182 (Figure 5). We note that the X-ray crystal structures do not reveal any detectable changes in intra-protein hydrogen bond lengths involving the hydrogen-bonded residues discussed here. Thus, the present results reflect the exquisite sensitivity of NMR chemical shifts to minute changes in molecular interactions and highlight the great complementarity of NMR and X-ray crystallography. Intriguingly, the present results, revealing a distinct correlation between the chemical shifts and enthalpy of binding, suggest that the chemical shift changes report on subtle structural rearrangements that modify interaction strengths both within the protein and between the protein and ligand in response to halogen substitutions, similar to previous conclusions for the specific case of CH-p interactions.^30^ Our present results provide a qualitative view for comparison with QM-calculated interaction energies (see later in discussion) and should motivate future studies including quantum-chemical calculations of chemical shift perturbations.

### ^15^N relaxation rate constants indicate minimal perturbations of protein dynamics

To investigate whether the halogen substituents differentially affect the conformational fluctuations of the protein, we measured ^15^N *R*_1_ relaxation rate constants on the samples resulting from the ITC titrations. To a first approximation, valid for HN groups experiencing limited fluctuation amplitudes on picosecond time scales, the ^15^N *R*_1_ relaxation rate is proportional to the order parameter,^31^^,^^32^ which varies from 0, for an H–N bond vector that samples all possible orientations on a timescale faster than the correlation time for the overall rotational diffusion of the protein, to 1, for a completely rigid H–N bond vector. The order parameter is turn is related to the conformational entropy of the protein backbone.^33^^,^^34^^,^^35^^,^^36^ The profile of *R*_1_ relaxation rate constants along the protein sequence reveals a high degree of similarity between the different complexes, with deviations occurring for only a few residues (Figure S6). The resulting mean values of *R*_1_ for the five complexes are indistinguishable, as determined from unbalanced one-way ANOVA test (*p* = 0.90 using unweighted data or *p* = 0.32 using data weighted by their estimated uncertainties; Figure S6 shows a boxplot). Thus, we conclude that the different complexes do not differ in any major way in their dynamics or the associated conformational entropy.

### Quantum mechanical interaction energies and solvation-free energies correlate with the experimental free energy of ligand binding

**Figure 7:**
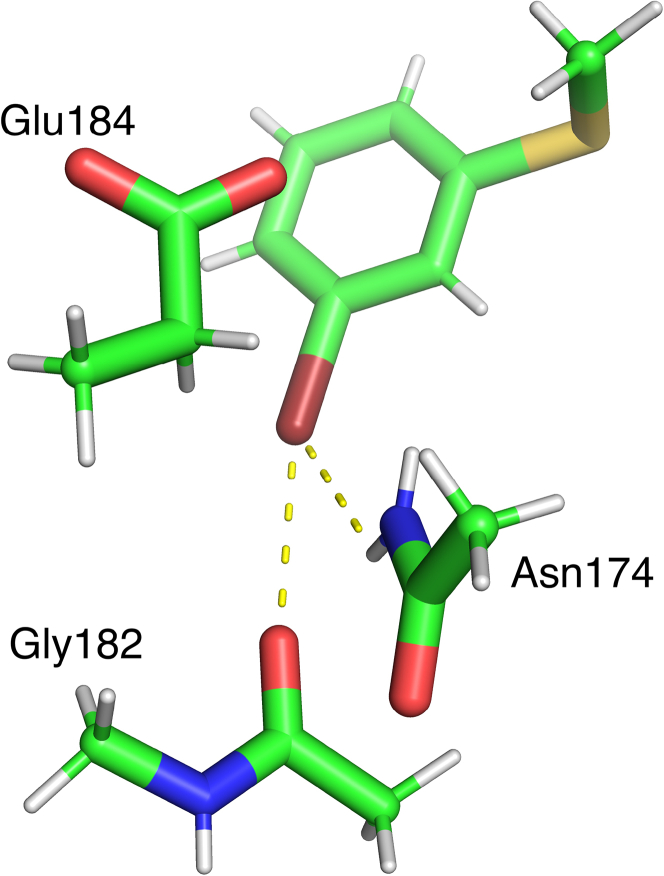
Model system used in the QM calculations Atoms included in model systems used to calculate interaction energies between halobenzene ligand surrogates and three nearby residues (Asn174, Gly182, and Glu184), as exemplified with the **Br** complex.

To further explain the observed binding enthalpies, we performed QM calculations of the interaction energies between the surrounding residues and the variable part of the ligands, using *m*-halo methyl(phenyl)sulfanes as ligand surrogates (Figure 7). The geometries of the calculations were based on the crystal structures and include only those residues that are in direct contact with the halogen moiety. In addition, we calculated the solvation-free energy of the five unbound ligands **H**, **F**, **Cl**, **Br**, and **I** with the COSMO-RS approach,^37^^,^^38^ as a crude estimate of the desolvation free energy of the ligand surrogate structures upon binding to the protein.

**Figure 8:**
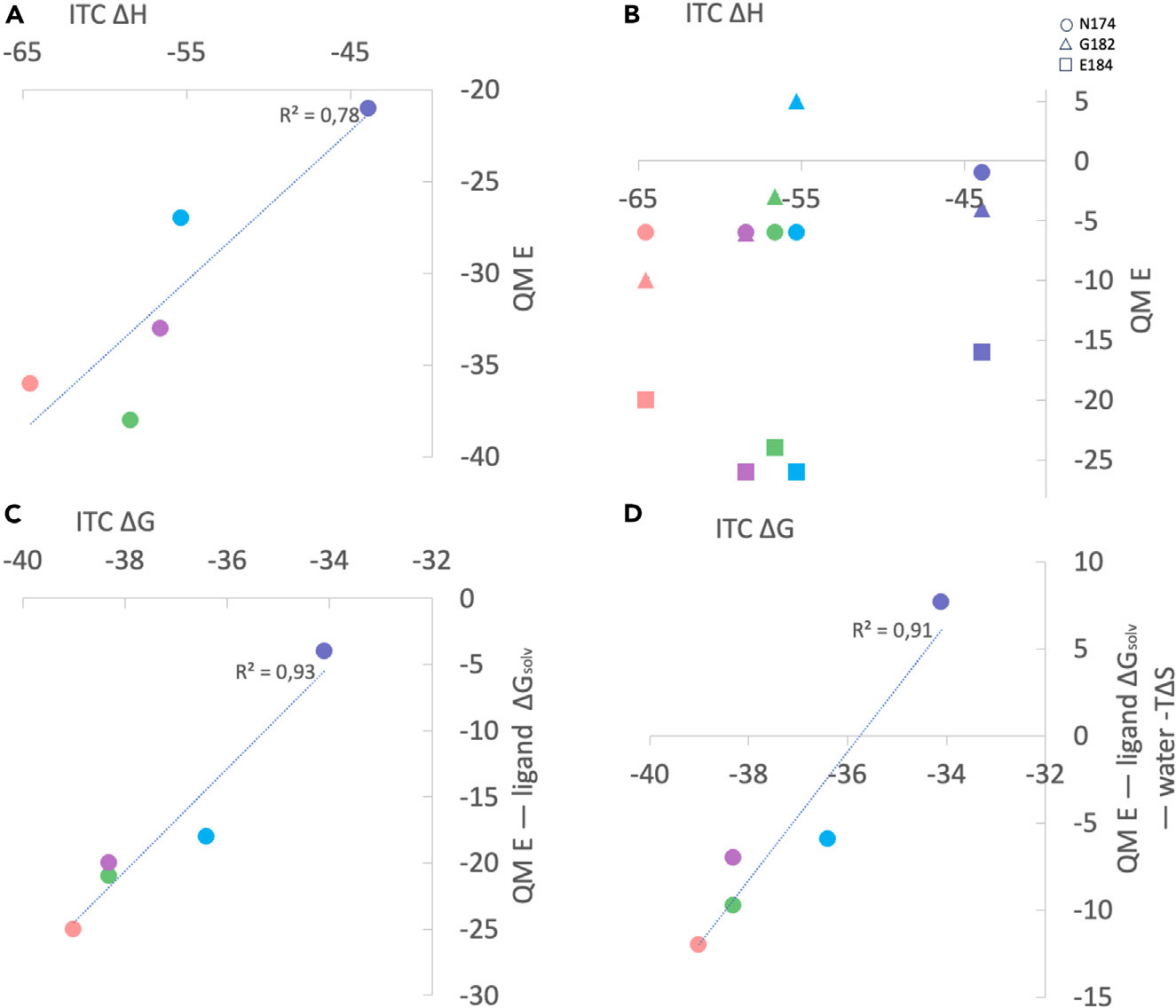
Comparison of experimental and calculated thermodynamic parameters Δ*H* and Δ*G* for the five ligands H, F, Cl, Br, and I (A) Comparison of the experimental Δ*H* from ITC with the sum of QM interaction energies between the ligands and the three nearby residues. (B) Comparison of the experimental Δ*H* from ITC with the individual QM interaction energies between the ligands and each of three nearby residues (Asn174, circle; Gly182, triangle; Glu184, square). (C) Comparison of the experimental Δ*G* from ITC with the calculated Δ*G*, estimated as Sum – Solv (see Table 2). (D) Comparison of the experimental Δ*G* from ITC with the calculated Δ*G*, estimated as Sum – Solv – GIST entropies (Table 3). All energies are in kJ/mol. The symbols are color coded: **H** (slate), **F** (cyan), **Cl** (magenta), **Br** (green), and **I** (salmon pink).

The interaction energy with Gly182 follows the expected trend for the four halogenated ligands, i.e., a more favorable energy with the larger halogens, reflecting the XB between the halogen σ-hole and the carbonyl oxygen of Gly182 (Table 2; Figures 8A and 8B). However, the **H** ligand breaks the trend, having an interaction energy similar to that of the **Cl** ligand. The hydrogen atoms on the phenyl ring possess slightly positive character and thus have an electrostatic complementarity to the Gly182 oxygen (as have the halogens Cl, Br, and I), whereas the F atom does not, and therefore has an unfavorable interaction energy with the Gly182 oxygen. The same trend is found for the ligand solvation free energies, which become more favorable when going from **F** to **I** (−10 to −17 kJ/mol). Experimental solvation free energies of *m*-halobenzenes also follow a similar trend: −3.3, −4.7, and −6.1 kJ/mol for F, Cl, Br.^39^ These results reflect how the σ-hole increases the ligand hydrophilicity with increasing halogen size. However, the **H** ligand experiences a solvation free energy almost as favorable (−16 kJ/mol) as the **I** ligand.

**Table 2:** QM interaction energies and ligand solvation free energies^a^

	Solv	Asn174	Gly182	Glu184	Sum
**H**	−16	−1	−4	−16	−4
**F**	−10	−6	5	−26	−18
**Cl**	−13	−6	−3	−24	−21
**Br**	−14	−6	−6	−26	−25
**I**	−17	−6	−10	−20	−20

a QM interaction energies are listed for each of the three ligand–residue pairs together with solvation free energies for the ligands (Solv), as well as the sum of the three interaction energies minus the ligand solvation free energies (Sum). All energies are in kJ/mol.

The interaction energy with the Asn174 side chain is essentially identical for the four halogen-containing ligands, but less favorable for the **H** ligand. This reflects the hydrogen bond from the side chain amide group of Asn174 to the electronegative belt of the ligand halogen atoms. **H** cannot accept a hydrogen bond and gives a CH···HN distance of 3.5 Å. The interaction energies with the Glu184 side chain are most favorable for the ligands with **F**, **Cl,** and **Br** (−24 to −26 kJ/mol), whereas those with **I** and especially **H** are less favorable (−20 and −16 kJ/mol, respectively). These values correlate with the distances between the hydrogen or halogen and the closest atom of Glu184 (3.2, 2.8, 2.9, 3.0, and 3.2 Å for **H**, **F**, **Cl**, **Br**, and **I**, respectively, giving a correlation of 0.72 to the binding energies) and also the distance between the ligand C—X carbon atom and the Gly182 carbonyl oxygen (4.3, 3.4, 3.5, 3.6, and 4.1 Å for **H**, **F**, **Cl**, **Br**, and **I**, respectively, giving a correlation of 0.91 to the binding energies).The deviation of the **I**—Glu184 interaction energy from the trend of **F** to **Br** may be a consequence of increased steric crowding of **I** as observed in a study of XB in DNA junctions.^10^

The sum of the three interaction energies involving Asn174, Gly182, and Glu184 provides an approximate estimate of the interaction enthalpies of the ligands, which reasonably follows the trend in experimental Δ*H* determined by ITC (Figure 8A). Furthermore, by subtracting the solvation free energy of the ligand (*i.e*., an approximation of the desolvation free energies of the binding) from the interaction energies, we obtain an estimate of the difference in total interaction free energies (Figure 8C). Notably, these differences correlate well with the trend observed in the experimental Δ*G* from ITC (R = 0.93). Furthermore, we retain a good correlation with the experimental Δ*G* from ITC when including in the estimated interaction free energies the entropy of the water molecules observed near the halogen in the X-ray structures, as estimated by GIST analysis of MD simulations (see later in discussion) (Figure 8D, *R* = 0.91). Hence, the binding free energies of the five ligands are likely composed of several factors, each of which shows an individual trend. We recognize that a number of effects remain unaccounted for in these calculations, e.g., changes in intra-protein hydrogen bonds detected by NMR. Nevertheless, the ligand–protein interactions near the halogen atom combined with the ligand solvation free energies appear to capture the central contributions to the variation in ligand binding thermodynamics involving XB in galectin-3.

### Molecular dynamics simulations and grid inhomogeneous solvation theory analysis reveal similar water entropy across the halogen bonding complexes

**Figure 9:**
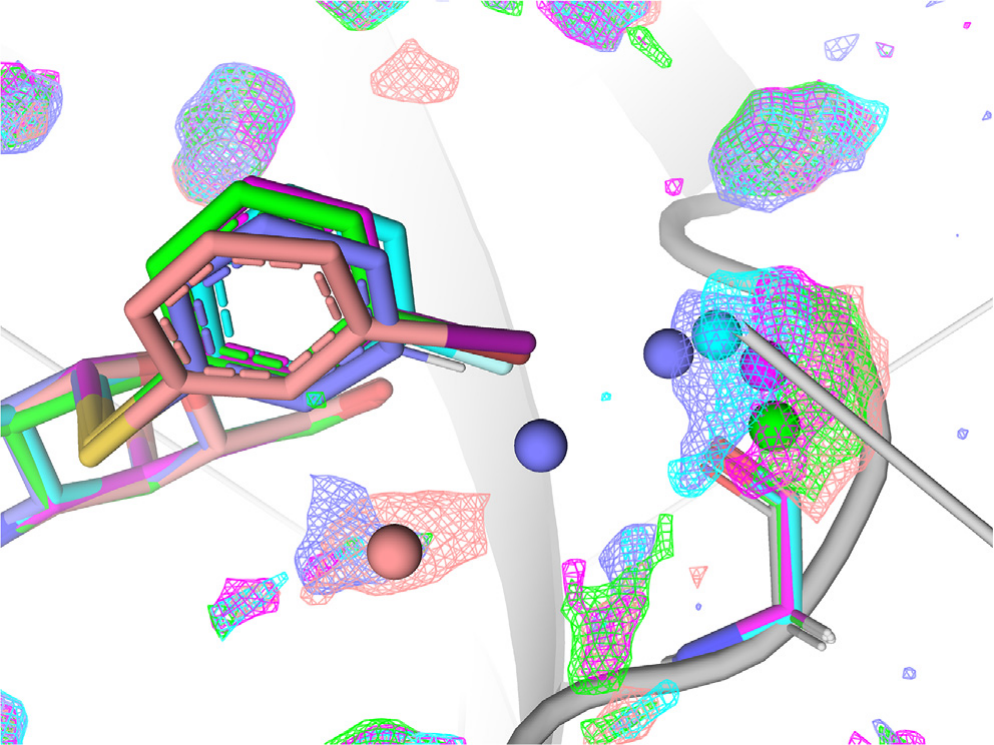
Superposition of the crystal structures and the water densities from the MD simulations for the five ligand–galectin-3C complexes, focused on the variable part of the ligands The isodensity level is five times the bulk density. The variable water molecules in the crystal structures are shown as balls. The structures and densities are color coded: **H** (slate), **F** (cyan), **Cl** (magenta), **Br** (green), and **I** (salmon pink).

We ran a set of 10 × 10 ns simulations for each of the five ligand–galectin-3C complexes and then performed GIST thermodynamic analysis of water sites surrounding the ligand. The ligand aglycon phenyl does not rotate and its halogen substituent remains in proximity to the Gly182 oxygen during the simulations. The water sites observed in the crystal structures are partly reproduced in the simulations. In all cases, the water molecules are exchanging rapidly with the bulk solvent, similar to previous results for other galectin-3C complexes (Figure 9).^40^ Most of the strongly populated water sites are identical in the five simulations, but the water site closest to the variable halogen atom shows a significant variation in population density depending on the ligand. This water moves away from the halogen atom as the halogen radius increases. The water densities are somewhat further away from the ligand than what is observed for the crystal-water molecules, which may reflect the reduced dynamics at 100 K, the temperature used in the crystal diffraction experiments. However, the trend is accurately reproduced. The only difference is that the simulations show high water density also near the **I** atom. In principle, this could be due to the lower resolution of the **I** complex (1.34 Å) compared to the other cases (all ∼1.1 Å). However, a relatively well-ordered water molecule should be visible even at 1.35 Å. A more likely explanation is that the force field used in the MD simulations cannot accurately model the σ-hole of the heavier halogens. Instead, the variable atom has rather low charge, which represents a spatial average over the positive and negative potentials around the halogen atoms and likely gives a poor description of the actual potential at each point around the atom. The net charge is positive for **H** (0.15 *e*), negative for the three first halogen atoms with a decreasing trend (−0.23 *e* for **F**, −0.17 *e* for **Cl,** and −0.07 *e* for **Br**) and slightly positive again for **I** (0.04 *e*); we return to this issue later in discussion. However, the simulations reproduce the other water sites in the structure of the **I** complex, including the water molecule (salmon pink in Figure 9) that is observed only in the crystal structure of the **I** complex (Figure 9).

**Figure 10:**
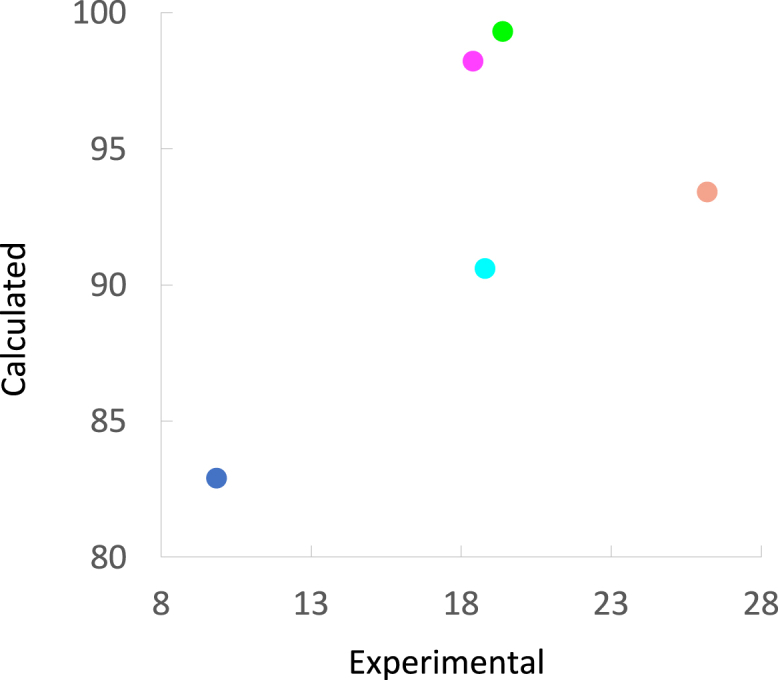
Correlation between the calculated water –*T*Δ*S*_tot_ and the experimental total –*T*Δ*S*_ITC_ (kJ/mol) The markers are color coded: **H** (slate), **F** (cyan), **Cl** (magenta), **Br** (green), and **I** (salmon pink).

Water entropies from the GIST analyses partly follow the experimental trends observed for the total ITC entropies. Analysis of the total entropy for the water molecules located near the ligand (–*T*Δ*S*_tot_ in Table 3) reveals that the entropy is lowest for the complex with the **H** ligand, in accordance with the entropy measure by ITC (Table 1; Figure 10). The –*T*Δ*S*_tot_ values of the complexes with the **F**, **Cl**, and **Br** ligands are appreciably greater, by 8–16 kJ/mol, reproducing the increase in ITC entropy of 9–10 kJ/mol upon going from **H** to **F**, **Cl**, or **Br**. However, for the **I** ligand complex, the –*T*Δ*S*_tot_ value is only 3 kJ/mol greater than that for the **F** ligand complex, and thus lower than the values for the **Cl** and **Br** ligand complexes (by 5–6 kJ/mol). For comparison, the ITC entropy increases by 7–8 kJ/mol upon going from **F**, **Cl**, or **Br** to **I** (Figure 10). Restricting the GIST entropy calculations to the water molecule(s) closest in the X-ray structures to the variable ligand atom (Table 3), shows that the trends do not originate from this water molecule. For this region, the five simulations give essentially the same entropy (11.3–13.0 kJ/mol) within the statistical uncertainty (±0.4–0.8 kJ/mol). Consequently, the difference in binding entropy obtained by GIST analysis is not primarily related to this particular water molecule, but rather to the water probability density across the entire binding site. It has been observed that water molecules placed near polyamphiphilic environments, such as a lectin ligand-binding site, have higher entropy than in the bulk phase. In contrast, water molecules in the bulk phase experience a more well-coordinated hydrogen bond network leading to comparatively lower entropy.^41^^,^^42^ Similarly, the solvation of the unbound **I** ligand likely involves poorly coordinated water molecules surrounding the larger iodine atom. One may speculate that binding to galectin-3 displaces most of these water molecules, releasing them to bulk solvent where the more well-coordinated hydrogen bonding network leads to favorable solvation enthalpy counteracted by a largely unfavorable solvation entropy. In contrast, the **Cl** and **Br** ligands emerge as the halogens of choice due to more favorable solvation thermodynamics, despite having weaker XB interaction with Gly182 than does **I**. This observation resembles an earlier case of XB in a protein complex in which bromine was favored over iodine, despite the iodo-XB being enthalpically favored.^10^ However, those authors suggested the unfavorable binding entropy of the iodine compound was due to steric crowding in the interaction site.

**Table 3:** Water entropy (–*T*Δ*S* in units of kJ/mol) from GIST analysis of the five galectin-3C–ligand complexes

Ligand	–*T*∆*S*_tot_	–*T*∆*S*	–*T*∆*S*
	binding site	water (from each structure)	water (from Br-structure)
**H**	82.9 ± 0.8^a^	11.7 ± 0.2, 12.5 ± 0.2	12.2 ± 0.2
**F**	90.6 ± 0.6	12.1 ± 0.1	12.9 ± 0.1
**Cl**	98.2 ± 0.3	11.3 ± 0.2	13.1 ± 0.2
**Br**	99.3 ± 0.6	13.0 ± 0.1	13.0 ± 0.1
**I**	93.3 ± 0.9	13.0 ± 0.3	10.2 ± 0.2

The GIST calculations included either all water sites in the entire binding site, or only the sites corresponding to the water molecule(s) observed in the crystal structures (two for H and one for the others), or only the water molecule observed in the Br-complex.

a Reported uncertainties are the standard errors over the ten independent MD simulations.

We investigated whether we could improve the representation of the electric potential of the halogen atoms in the force field. It has been suggested that an improved description of halogen atoms in molecular mechanics (MM) calculations can be obtained by using an extra point charge (EP), outside the halogen along the C–XB axis.^43^^,^^44^^,^^45^ Thereby, this charge would represent the positive σ-hole, whereas the halogen atom would have a band of negative charge, perpendicular to the C—X bond direction, that can accept hydrogen bonds. We calibrated the EP–halogen distances to QM calculations for halogen–backbone interaction energies and X ··· OC distances. Several other groups have used similar approaches, but they have suggested shorter distances between the EP and the halogen atom.^46^^,^^47^ Using such an approach in the MD simulations, we obtained calculated GIST entropies for the **Cl** and **Br** ligands close to those of **F** (Figure S4), in accordance with the measured entropies. However, at the same time, the calculated entropy for **I** decreased by 11 kJ/mol, falling off the correlation line of the other ligands. Moreover, the water densities around the ligands (Figure 9) moved away from the position of the crystal water molecules for all three ligands with the extra charge (**Cl**, **Br**, and **I** (Figure S5). Thus, our results show that the calculated entropies and the position of the water molecule are sensitive to the treatment of the halogen in the MM force field, but we were not able to find any parameters that satisfactorily reproduce both the experimentally observed structures and entropies. It is likely that a more accurate description of the halogen atom is needed, taking into account that the electron density is modified by surrounding atoms, breaking the symmetry so that the negative ring splits into regions of more or less negative charge.^48^^,^^49^^,^^50^ We also performed free-energy perturbation calculations of the relative binding affinities of the five ligands and showed that the standard parameters reproduced the experimental binding affinities excellently (within 1.8 kJ/mol), whereas the EP parameters gave much worse results (*cf*. Table S3).

### Conclusions

The XB is a well-established contributor to intermolecular interactions strengths in synthetic molecular systems used in e.g., material chemistry, supramolecular chemistry, and medicinal chemistry. While the XB is relatively well understood in terms of its physical and thermodynamic basis and its interplay with orthogonal hydrogen bonds, e.g., He-XB, the influence of XB on protein structure, dynamics, and solvation in ligand–protein binding sites is less well understood. The immunity- and cancer-related protein galectin-3 presents a rather shallow and amphiphilic ligand binding site that was recently targeted by small lead molecules exploiting a surface-exposed XB interaction to reach nM affinities.^16^^,^^51^^,^^52^ To understand the basis of the affinity enhancement induced by a solvent-exposed XB, we conducted an extensive structural and thermodynamic study of galectin-3 binding to a systematic series of ligands with increasing halogen size from **F** to **I**. All ligands, including the **H** control ligand, bind in nearly identical poses with their halophenyl rings showing only minor displacements that maintain a close interaction between the halogen and the carbonyl oxygen of Gly182, which keeps the XB distance almost constant from **F** to **I** (3.1–3.3 Å). Furthermore, The X-ray crystal structures reveal that a water molecule is successively pushed away by the size of the halogen, with an X–O distance that increases from 3.1 Å for **F** to 3.6 Å for **Br**, whereas the water molecule is completely displaced in the structure with **I**. ITC studies revealed that the increased halogen size from **H**, **F**, **Cl**, and **Br** to **I** gives rise to an increase in the binding enthalpy, which is compensated by unfavorable entropy changes. The entropy penalty is lowest for the **H** ligand and increases with **F, Cl,** and **Br** ligands and even more so for the enthalpically most favorable **I** ligand. NMR spectroscopy reveals that the experimental binding enthalpies correlate with chemical shift changes observed for residues located close to the halogen, suggesting that the chemical shift captures variations in interaction energies among the complexes that are not evident from the crystal structures. QM calculations show that the variation in binding energies among the five ligands **H, F, Cl, Br**, and **I** is governed mainly by the interaction with the carbonyl of Gly182 together with other nearby residues. The QM interaction energy between the ligand halophenyl and Gly182, as well as the ligand solvation free energy, is increasingly favorable from **F** to **I**. Further, the QM interaction energies largely correlate with the experimental binding enthalpies determined by ITC, which suggests that the XB contributes significantly to the binding. Intriguingly, the combination of QM interaction energies and ligand desolvation free energies correlate well with the free energy of binding as determined by ITC. Furthermore, the compensatory entropic penalties are proposed to also include contributions from different solvent reorganization thermodynamics for the various complexes. GIST results indicate little difference in the entropy of the water molecule(s) closest to the variable halogen atom. Instead, the GIST analysis shows that water sites across the entire binding site contribute to the solvation thermodynamics. Taken together, the present study shows a case in which solvation effects play a critical role in overall XB thermodynamics in ligands binding to a shallow and exposed protein binding site: The variation of enthalpy of complex formation is largely governed by the ligand halogen—Gly182 backbone carbonyl oxygen interaction, which together with the ligand solvation-free energy changes are the dominating contributing factors to the overall free energy of complex formation. Thus, the optimal choice of ligand halogen atom should be based on a carefully balanced consideration of both XB strength and solvation effects.

### Limitations of the study

One of the limitations of this study lies in that the accuracy in the overall thermodynamic parameters determined with ITC is close to the differences between different ligand complexes studied. Secondly, the X-ray analyses reveal the complex structures at 100K and not at ambient temperature. Furthermore, the ^15^N relaxation rate experiments only monitor a sub-set of protein bonds. The QM calculations on the halogen atom interactions are performed with only three nearby amino acid side chains, thus excluding any remote effects. Finally, the limited accuracy of the description of halogen bonds by the force fields used in the molecular dynamics simulations, and GRID analyses may, as discussed in the text, influence the results.

## STAR★Methods

### Key resources table

**Table:** 

REAGENT or RESOURCE	SOURCE	IDENTIFIER
**Chemicals, peptides, and recombinant proteins**		

**H**	Zetterberg et al.^16^	https://doi.org/10.1002/cmdc.201700744
**F**	This paper	–
**Cl**	Zetterberg et al.^16^	https://doi.org/10.1002/cmdc.201700744
**Br**	Zetterberg et al.^16^	https://doi.org/10.1002/cmdc.201700744
**I**	Zetterberg et al.^16^	https://doi.org/10.1002/cmdc.201700744
Galectin-3C	Diehl et al.,^18^ Diehl et al.^53^	https://doi.org/10.1007/s10858-009-9356-5, https://doi.org/10.1021/ja105852y

**Deposited data**		

Galectin-3C in complex with **H**	This paper	PDB: 6RZI
Galectin-3C in complex with **F**	This paper	PDB: 6RZJ
Galectin-3C in complex with **Cl**	This paper	PDB: 6RZK
Galectin-3C in complex with **Br**	This paper	PDB: 6RZI
Galectin-3C in complex with **I**	This paper	PDB: 6RZJ

**Software and algorithms**		

NIPTIC	Keller et al.^17^	https://doi.org/10.1038/nprot.2016.044
SEDPHAT	Brautigam et al.^54^	https://doi.org/10.1038/nprot.2016.044
ChemDraw Professional 21.0	PerkinElmer	https://www.perkinelmer.com/category/chemdraw
NMRPipe	Delaglio et al.^55^	https://doi.org/10.1007/BF00197809
CcpNmr	Vranken et al.^56^	https://doi.org/10.1002/prot.20449
Turbomole 7.2	Furche et al.^57^	https://www.turbomole.org
AMBER	Case et al.^58^	https://ambermd.org

### Resource availability

#### Lead contact

Further information and requests for resources and reagents should be directed to and will be fulfilled by the lead contact, Ulf J. Nilsson (ulf.nilsson@chem.lu.se).

#### Materials availability

This study did not generate new unique reagents.

#### Data and code availability

• The galectin-3C X-ray structural data reported in this paper have been deposited in the RCSB Protein Data Bank. The accession numbers for the public datasets used in this paper are listed in the key resources table.
• This paper does not report original code.
• Any additional information required to reanalyze the data reported in this paper is available from the lead contact upon request.

### Method details

#### Synthesis

##### General synthesis methods

^1^H and ^13^C NMR spectra were recorded on a Bruker Avance II 400 MHz spectrometer at ambient temperature; multiplicities are quoted as singlet (s), doublet (d), doublet of doublets (dd), triplet (t), apparent triplet (at) or apparent triplet of doublets (atd). NMR signals were assigned from COSY, HMQC and HMBC spectra. Chemical shifts are given in ppm downfield from the signal for Me_4_Si, with reference to residual CDCl_3_ or CD_3_OD at 7.26 and 3.31 ppm, respectively. Thin-layer chromatography (TLC) was carried out on Merck Kieselgel sheets, pre-coated with 60F254 silica. Plates were visualized using UV light and developed by charring with ethanolic H_2_SO_4_ (10%). Preparative chromatography was performed using silica gel (Acros Organics 40–60 μm, 60 Å) columns. DMF was distilled and stored over 4 Å M.S. Solvents were dried using an IT PS-Micro solvent dispenser and storing over activated M.S. Reagents were supplied by Sigma-Aldrich and used as-is. Purity and HRMS analysis were performed using UPLC/MS with UV/VIS detection on a Waters Acquity UPLC + Waters XEVO-G2 system using a Waters Acquity CSH C18, 1.7 μm, 2.1 ×100 mm column. Samples were run using a gradient with water (0.1% formic acid) and acetonitrile, at a flow rate of 0.50 mL/min and a column temperature 60°C. Gradient parameters: 0–0.7 min: 40% acetonitrile, 0.7–10.0 min: 40–99% acetonitrile, 10.0–11.0 min 99% acetonitrile, 11.0–11.1 min 99–40% acetonitrile, 11.1–13 min 40% acetonitrile, 3 or 6 μL injection, detection 190–300 nm. MS was performed with direct infusion using electrospray ionization (ESI). Cap voltage 3.0 kV, cone voltage 40 kV, ext 4, source temp 120°C, desolvation temp 500°C, cone gas 50, desolvation gas 800, centroid resolution mode, m/z interval 50–1200, lockspray. Calibration: Leu-Enkephalin m/z 556.2771, 0.25 s every 30 s, three reference scans were averaged for lock mass correction.

The **H**, **Cl**, **Br**, and **I** ligands have been described previously,^16^ and the **F** ligand was synthesized following essentially the same route.

##### 3-Fluorophenyl 2,4,6-tri-*O*-acetyl-3-azido-3-deoxy-1-thio-α-D-galactopyranoside

Sodium hydride (168.1 mg, 4.07 mmol, 60% in oil) and 3-fluorothiophenol (345 μL, 4.08 mmol) were dissolved in dry DMF (5 mL) at 0°C. To this solution was added freshly prepared 2,4,6-tri-*O*-acetyl-3-azido-3-deoxy-β-D-galactopyranosyl chloride (467 mg, 1.36 mmol) in dry DMF (5 mL).^16^ After 5 min of stirring, the reaction mixture was diluted with additional dry DMF (2 mL) and stirred at 50°C overnight. The reaction progress was monitored by TLC (1:1 heptane/EtOAc) and when complete, diluted with EtOAc (40 mL) and washed with brine (4 x 50 mL), water (15 mL), dried over Na_2_SO_4_, filtered, and concentrated. The crude (797 mg) was further purified by column chromatography (3:1 heptane/EtOAc) to give 3-fluorophenyl 2,4,6-tri-*O*-acetyl-3-azido-3-deoxy-1-thio-α-D-galactopyranoside as a white solid (329 mg, 55%). ^1^H NMR (400 MHz, CDCl_3_) δ 7.33–7.25 (m, 1H), 7.26–7.19 (m, 2H), 7.03–6.94 (m, 1H), 5.99 (d, *J =* 5.5 Hz, 1H), 5.47 (dd, *J =* 3.3, 1.2 Hz, 1H), 5.28 (dd, *J =* 11.0, 5.5 Hz, 1H), 4.69–4.61 (m, 1H), 4.13 (dd, *J =* 11.5, 4.9 Hz, 1H), 4.02 (dd, *J =* 11.6, 7.8 Hz, 1H), 3.95 (dd, *J =* 11.0, 3.4 Hz, 1H), 2.18 (s, 3H), 2.16 (s, 3H), 1.99 (s, 3H). ^13^C NMR (126 MHz, CDCl_3_) δ 170.37, 169.78, 169.74, 130.27, 130.20, 127.21, 118.68, 118.50, 114.87, 114.70, 84.88, 69.65, 67.99, 67.68, 61.98, 58.61, 20.73, 20.50, 20.44. HRMS calculated for [C_18_H_28_FN_3_O_7_S + Na^+^] 442.0904; found: 442.0910.

##### 3-Fluorophenyl 2,4,6-tri-*O*-acetyl-3-deoxy-3-[4-(3,4,5-trifluorophenyl)-1*H*-1,2,3-triazol-1-yl]-1-thio-α-D-galactopyranoside

3-Fluorophenyl 2,4,6-tri-*O*-acetyl-3-azido-3-deoxy-1-thio-α-D-galactopyranoside (325.5 mg, 0.737 mmol) was dissolved in dry acetonitrile (30 mL) at room temperature and CuI (70.2 mg, 0.369 mmol) was added. The reaction mixture flask was covered with aluminum foil and 3,4,5-trifluorophenylacetylene (222 μL, 1.47 mmol) was added and left stirring for 5 min at room temperature. Then, DIPEA (128 μL, 0.737 mmol) was slowly added to the reaction mixture and heated up to 45°C. Two hours later, the temperature was increased to 60°C for 90 min and then left to drop to 30°C overnight. The reaction was monitored by TLC (1:1 heptane/EtOAc). After 23 h, the mixture was filtered through a short silica column in EtOAc to afford 3-fluorophenyl 2,4,6-tri-*O*-acetyl-3-deoxy-3-[4-(3,4,5-trifluorophenyl)-1*H*-1,2,3-triazol-1-yl]-1-thio-α-D-galactopyranoside (326 mg, 74% yield). ^1^H NMR (400 MHz, CDCl_3_) δ 7.79 (s, 1H), 7.44 (t, *J =* 7.2 Hz, 2H), 7.37–7.27 (m, 3H), 7.08–6.98 (m, 1H), 6.16 (d, *J =* 5.5 Hz, 1H), 6.08 (dd, *J =* 11.6, 5.6 Hz, 1H), 5.61 (d, *J =* 2.2 Hz, 1H), 5.22 (dd, *J =* 11.6, 2.9 Hz, 1H), 4.88 (dd, *J =* 6.5, 6.0 Hz, 1H), 4.16 (dd, *J =* 11.6, 5.2 Hz, 1H), 4.08 (dd, *J =* 11.6, 7.5 Hz, 1H), 2.06 (s, 3H), 2.00 (s, 3H), 1.97 (s, 3H). ^13^C NMR (126 MHz, CDCl_3_) δ 170.33, 169.49, 169.11, 130.44, 127.52, 118.95, 118.77, 115.20, 115.03, 109.92, 109.75, 85.05, 68.68, 67.60, 66.73, 61.77, 59.21, 20.48, 20.42, 20.32. HRMS calculated for [C_26_H_24_F_4_N_3_O_7_S]^+^ 598.1271; found: 598.1259.

##### 3-Fluorophenyl 3-deoxy-3-[4-(3,4,5-trifluorophenyl)-1*H*-1,2,3-triazol-1-yl]-1-thio-**α**-D-galactopyranoside (F)

3-Fluorophenyl 2,4,6-tri-*O*-acetyl-3-deoxy-3-[4-(3,4,5-trifluorophenyl)-1*H*-1,2,3-triazol-1-yl]-1-thio-α-D-galactopyranoside (30.8 mg, 0.051 mmol) was dissolved in a solution of NaOMe (54 mg, 0.206 mmol) in MeOH (2 mL). The reaction was monitored on TLC until the disappearance of the starting material (4:1 DCM/EtOAc). The reaction was quenched after 30 min by adding Amberlite IR-120 (H^+^) until neutral pH. The solvent was reduced under vacuum and the crude was purified with preparative HPLC on Agilent 1260 Infinity (pump, injection, UV detector at 210 and 254 nm), column SymmetryPrep C18 (5 μm, 19 × 100 mm in diameter per length), gradient mobile phase from 10% to 100% acetonitrile (in water, 0.1% formic acid) over 22 min to afford **F** (11.6 mg, 64% yield). ^1^H NMR (400 MHz, CD_3_OD) δ 8.55 (s, 1H), 7.66 (dd, *J =* 9.0, 6.6 Hz, 2H), 7.41 (ddt, *J =* 6.6, 3.9, 1.2 Hz, 2H), 7.34 (td, *J =* 8.0, 5.9 Hz, 1H), 7.03 (tdd, *J =* 8.7, 2.5, 1.1 Hz, 1H), 5.86 (d, *J =* 5.2 Hz, 1H), 4.99 (dd, *J =* 11.5, 2.8 Hz, 1H), 4.91 (dd, *J =* 11.5, 5.2 Hz, 1H), 4.51 (t, *J =* 6.4 Hz, 1H), 4.20 (dd, *J =* 2.7, 1.0 Hz, 1H), 3.71 (qd, *J =* 11.4, 6.2 Hz, 2H); ^13^C NMR (101 MHz, CD_3_OD) δ 166.61, 165.37, 162.91, 137.90, 131.49, 131.41, 128.91, 122.99, 119.72, 119.49, 115.25, 115.04, 110.90, 110.67, 91.21, 73.28, 69.63, 66.74, 65.58, 62.06. HRMS calculated for [C_20_H_18_F_4_N_3_O_4_S]^+^ 472.0954; found: 472.0951. Analytical purity was 99.9%.

#### Crystallization and data collection

The C-terminal carbohydrate-recognition domain of galectin-3 (galectin-3C) was expressed and purified following published protocols,^18^^,^^53^ yielding a protein stock solution of 19.2 mg/mL in ME-PBS buffer (10 mM Na_2_HPO_4_, 1.8 mM KH_2_PO_4_, 137 mM NaCl, 2.7 mM KCl, pH 7.5, 1 mM ethylenediaminetetraacetic acid (EDTA), 5 mM β-mercaptoethanol). Ligands were dissolved in dimethyl sulfoxide (DMSO) to 100 mM stock solutions. The protein (9 μL) was mixed with ligand (1 μL) and incubated on ice for 1 h. The solution was centrifuged at 10,000 rpm, 4°C for 10 min to remove any insoluble ligand. A 2 + 2 μL hanging drop was set up over 0.5 mL reservoir solution (20% PEG 4000, 0.1 M Tris/HCl pH 7.5, 0.4 M NaSCN, 8 mM β-mercaptoethanol). The drop was then seeded with apo-galectin-3C crystal seeds. Crystals appeared within 2 days and were fully grown within a week. Crystals were flash-frozen in cryoprotectant solution (15% PEG 400, 25.5 w/v % PEG 4000, 0.25 M NaSCN, 85 mM Tris/HCl pH 7.5, 8 mM β-mercaptoethanol). Data for all the compounds were collected at 100 K at the BioMAX beamline of the MAX IV synchrotron, Lund, Sweden.^59^ Data for all structures were integrated using XDS^60^ and scaled using AIMLESS from the CCP4 package.^61^ The structures were determined by using the apo structure of galectin-3C, PDB ID 3ZSL,^40^ determined to 1.08 Å resolution, as starting model. Molecular replacement was done with Phaser^62^ in Phenix.^63^ Restraints for all ligands were generated using eLBOW^64^ from Phenix. All refinement was done using phenix.refine.^65^ Further model building and manipulations were done in Coot.^66^ Figures were generated using PyMOL (Schrodinger, LLC).

#### Isothermal titration calorimetry

Galectin-3C uniformly labeled with ^15^N was prepared by extensive dialysis against 5 mM 4-(2-hydroxyethyl)-1-piperazinethanesulfonic acid (HEPES) buffer to remove all lactose, followed by centrifugation at 14 × 10^3^ rpm to remove any aggregates. All ligands were dissolved to 5 mM in stock solutions in DMSO. Isothermal titration calorimetry (ITC) experiments were performed on a MicroCal PEAQ–ITC instrument (Malvern Panalytical Ltd) at a temperature of 301 K by titrating the protein at a concentration of 287 μM into the cell (200 μL) containing the ligand at a concentration of 28.7 μM. The experiments were performed in this manner because of the low ligand solubility in buffer, which prevented preparation of samples with sufficiently high ligand concentrations to be used as injectant. The DMSO concentrations in the cell and the syringe were carefully matched to minimize the heat of dilution. Three replicate experiments (15 injections, spacing 240 s, 2.5 μL injection volume, 0.4 μL first injection) were performed for each complex. Peak integration was done using NITPIC.^17^

#### NMR sample preparation, experiments and data analysis

For each ligand–galectin-3C complex, an NMR sample was prepared by pooling the ITC cell content from two experiments, which adds up to 480 μL, and adding 10% D_2_O to yield a final protein concentration of approximately 40 μM. NMR experiments were performed at a temperature of 301 ± 0.1 K on a 600 MHz Varian/Agilent VNMRS DirectDrive spectrometer equipped with a ^1^H/^13^C/^15^N triple resonance room temperature probe. Sensitivity enhanced ^1^H-^15^N HSQC experiments^67^^,^^68^ were performed using 240 transients and spectral widths of 14 ppm (^1^H) and 32 ppm (^15^N), covered by 1024 and 128 points, respectively, resulting in an total experiment time of about 22 h per complex.

^15^N *R*_1_ rate constants were measured using the accordion-NUS (non-uniform sampling) experiment,^69^ as a combination of forward and reverse accordion modes (i.e., incrementing or decrementing the relaxation delay in step with *t*_1_). The NUS sampling schemes sampled 63 out of 125 points in the ^15^N dimension. One and the same sampling scheme was used for all forward experiments and another sampling scheme was used for all reverse experiments, on all 5 ligand–protein complexes. These two sampling schemes were optimized individually by generating 1000 sine-weighted Poisson-Gap sampling schemes from a previously published *R*_1_-accordion dataset acquired on galectin-3C and selecting the best scheme by comparing all generated schemes against the Cramér-Rao lower bound (CRLB).^69^ The ^1^H (^15^N) dimension was acquired with a spectral width of 8013 Hz (2100 Hz), sampled over 1920 (63) complex points. The forward and reverse accordion experiments were acquired in an interleaved fashion. The data were recorded in 3–4 individual experiments that were co-added prior to analysis. The total number of transients acquired for each complex (**H**, **F**, **Cl**, **Br**, and **I**) was (1888, 1120, 1888, 1864, and 1600), resulting in total acquisition times of 8–12 days.

The HSQC spectra were processed using NMRPipe,^55^ including a solvent filter, square cosine apodization, linear prediction in the indirect dimension, and zero filling to twice the number of points in both dimensions. All spectra were analyzed using the CcpNmr program suite^56^ to monitor chemical shift changes between complexes. Accordion-NUS data were analyzed as described using the spare reconstruction technique DSURE (damped super-resolution estimator) to model interferograms (i.e., data series along the indirect dimension *t*_1_) as sums of exponentially decaying sinusoidal signals^69^:

$$S\left(t\right)=\sum_{k}^{K}S_{k}\;\exp\;\left[i\omega_{k}t-R_{k}t\right]+\varepsilon \left(t\right)$$

where *S*_k_, *w*_k_, and *R*_k_ denote the complex-valued amplitude, frequency, and relaxation rate constant of the *k*th signal, respectively, and *e*(*t*) represents additive noise, and the sum runs over all *K* signals identified in a given interferogram. Reconstruction of the reverse mode accordion-NUS data involved inverting and complex conjugating the time domain data prior to estimation by DSURE. The DSURE algorithm was implemented in MATLAB (The Mathworks).

Residue-specific backbone amide chemical shift assignments were based on previous assignments of similar galectin-3C complexes,^18^ and aided by monitoring progressive chemical shift changes of individual backbone amides between spectra of different ligand–galectin-3C complexes.

#### Quantum-mechanical calculations

We employed quantum-mechanical (QM) calculations to obtain energies that can help to explain the experimentally observed differences in binding affinity of the studied compounds to galectin-3C (standard molecular mechanics force fields cannot properly describe XB and the σ-hole^45^^,^^70^). We used the crystal structures determined herein to calculate QM interaction energies between ligands and nearby protein residues, and to calculate solvation free energies for the ligands. All QM calculations were performed with the Turbomole 7.2 software.^57^^,^^71^

The interaction energy between each ligand and three nearby residues, Asn174, Gly182 and Glu184, was calculated using small model systems. The amide group of Gly182 was modeled as CH_3_–CO–NH–CH_3_ and the side chains of Asn174 and Glu184 were modeled as CH_3_–CO–NH_2_ and CH_3_–CH_2_–COO^–^, respectively. The interaction energy was calculated separately for each ligand–residue pair, using the TPSS-D3/def2-TZVP level of theory.^72^^,^^73^^,^^74^ Three single-point calculations were performed for each pair: one for the ligand (*E*_lig_), one for the residue (*E*_res_), and one for the complex (*E*_comp_). The interaction energy was then calculated as:

$$\Delta E=E_{\text{comp}}\;–\;E_{\text{res}}\;–E_{\text{lig}}$$

In addition, we calculated solvation free energies for the isolated ligands using the conductor-like screening model for real solvents (COSMO-RS),^37^^,^^38^ with the dielectric constant for water ε_r_ = 80, and optimized radii for all atoms.^75^ For each ligand, the solvation free energy was calculated based on two single-point calculations at the BP-D3/TZVP level of theory^74^^,^^76^^,^^77^^,^^78^ (one in vacuum and one in a continuum solvent with an infinite dielectric constant), as is required by the method. The solvation free energy of the isolated ligand gives an estimate of the desolvation penalty of each ligand.

#### MD simulations and solvation thermodynamics

All MD simulations were run with the AMBER software suite.^58^ The X-ray crystal structures obtained in this study were used as the starting points for the MD simulations. All water molecules from the crystal structures were kept in the simulations. Each galectin-3C complex was solvated in an octahedral box of water molecules extending at least 10 Å from the protein using the tleap module. The simulations were set up in the same way as in our previous studies of galectin-3C.^19^^,^^53^^,^^79^^,^^80^ All Glu and Asp residues were assumed to be negatively charged and all Lys and Arg residues positively charged, whereas the other residues were neutral. The active-site residue His158 was protonated on the ND1 atom, whereas the other three His residues were protonated on the NE2 atom, in accordance with the neutron structure of the lactose-bound state,^81^ NMR measurements,^81^ and previous extensive test calculations with MD.^82^ This resulted in a net charge of +4 for the protein. No counter ions were used in the simulations.

The protein was described by the AMBER ff14SB force field,^83^ water molecules with the TIP4P-Ewald model,^84^ whereas the ligands were treated with the general AMBER force field.^85^ Charges for the ligands were obtained with the restrained electrostatic potential method.^86^ The ligands were optimized with the semiempirical AM1 method,^87^ followed by a single-point calculation at the Hartree–Fock/6-31G∗ level to obtain the electrostatic potentials, sampled with the Merz–Kollman scheme.^88^ These calculations were performed with the Gaussian 09 software.^89^ The potentials were then used by antechamber to calculate the charges. Charges and atom types of the ligands are listed in the supplementary material.

For each complex, 10 × 10^3^ steps of minimization were used, followed by 20 ps constant-volume equilibration and 20 ps constant-pressure equilibration. Finally, the system was equilibrated for 1 ns, followed by 10 ns of production simulation, both performed with constant pressure. The GIST method requires snapshots from MD simulations in which the solute is kept restrained. Therefore, all MD simulations were performed with heavy non-water atoms restrained toward the starting crystal structure with a force constant of 209 kJ/mol/Å^2^. For each protein–ligand complex, 10 independent simulations were run, employing different solvation boxes and different starting velocities.^90^ Consequently, the total simulation time for each complex was 100 ns. All bonds involving hydrogen atoms were constrained to the equilibrium value using the SHAKE algorithm,^91^ allowing for a time step of 2 ps. The temperature was kept constant at 300 K using Langevin dynamics,^92^ with a collision frequency of 2 ps^−1^. The pressure was kept constant at 1 atm using a weak-coupling isotropic algorithm^93^ with a relaxation time of 1 ps. Long-range electrostatics were handled by particle-mesh Ewald summation^94^ with a fourth-order B spline interpolation and a tolerance of 10^−5^. The cut-off radius for Lennard-Jones interactions between atoms of neighboring boxes was set to 8 Å.

The structure and thermodynamics of the solvent around the five ligands bound to galectin-3C were analyzed with GIST^95^ implemented in the cpptraj module of the AMBER software.^58^ For each protein–ligand system, the water–water interaction energy, *E*_ww_, and solute–water interaction energy, *E*_sw_, as well as translational, *S*_trans_, and rotational, *S*_rot_, entropy contributions were calculated for a rectangular grid of dimensions 16 Å × 17 Å × 14 Å, centered on the ligand and extended at least 3 Å on each side of the ligand. We also calculated GIST enthalpy and entropy for the water molecule closest to the varying atom of the ligand using a cubic grid of 6 Å × 6 Å × 6 Å centered on the water oxygen atom (in the crystal structure). The grid was divided into cubic boxes (0.5 Å × 0.5 Å × 0.5 Å), for which the thermodynamic properties were calculated. The sum of these properties over the entire region reveals the changes in the hydration thermodynamics of the region for each cluster, relative to the thermodynamics of the bulk water.

#### Calculations with an extra point charge model for the halogens

We calibrated an extra point charge (EP) model for the **Cl**, **Br**, and **I** halogen atoms (*i.e*., the halogens with an σ-hole), following a published procedure.^43^^,^^44^^,^^45^ We used the same values for all MM parameters as in those studies, but we performed a calibration of the X–EP distance using QM calculations with *m*-halogenated methyl phenyl sulfides (as ligand surrogates) X-bonded to the carbonyl of *N*-methyl acetamide, optimized at the TPSS/def2-TZVPD level and with single-point energies calculated at the B3LYP/def2-QZVPD level (Table S2). Based on these results, X–EP distances of 1.95, 2.02 and 2.15 Å for **Cl**, **Br**, and **I**, respectively, were employed. These distances were selected to give a trend following the van der Waals radii of the three halogens, 1.75, 1.85, and 1.98 Å. With these parameters, we reproduce the QM interaction energies within 1–2 kJ/mol and the X–OC distances within 0.02–0.09 Å.

In addition, we performed similar QM calculations of the ligand surrogates and a water molecule to investigate how well water interactions are modeled. The data in Table S2 reveals that these interactions are relatively independent of the X–EP distances (within 1–2 kJ/mol). However, the MM models always underestimate the XB interaction energies by 6–7 kJ/mol and the X–OH_2_ distances are 0.21–0.25 Å too short. This illustrates the problem to model the anisotropic charge distribution of the halogens with a simple point-charge MM model.

We ran MD simulations for the optimized X–EP distance, as well as for five other X–EP distances, viz. the optimum values + 0.2 Å or −0.2, −0.4, −0.6 and −1.2 Å. The correlation between the calculated (GIST) and experimental entropies are shown in Figure S4 (the corresponding results with standard AMBER parameters are in Figure 10 in the main article). The calculated –*T*Δ*S*_tot_ values in general decrease with the X–EP distance, so that with the optimum values the results of Cl and Br fall close to F (light blue lying triangles), but the entropy of the I ligand decreased even more, so that it falls off the correlation line of the other ligands. It should be noted that only the calculation with optimum X–EP distances involve ten independent MD simulations, whereas the other are based on a single 10 ns simulation, which explains the sometimes somewhat unclear trends (the –*T*Δ*S*_tot_ values may vary by up to 8 kJ/mol for the individual simulations).

Based on the MD simulations, we calculated water densities for the five ligands (the corresponding results with the standard AMBER parameters are in Figure 9 in the main text). Unfortunately, it turned out that in all simulations with the EP model, the water densities have moved away from the position of the water molecule observed in the crystal structures as can be seen in Figure S5. In most cases, the water density moves somewhat closer to the crystal water molecule as the X–EP distance is decreased, but even with the shortest distance, the density is still quite far from the crystal water molecule.

We also performed free-energy perturbation (FEP) calculations of the binding affinity calculations of the **H, Cl, Br** and **I** ligands, relative to that of the **F** ligand, both without and with the EP parameters. The results in Table S3 show that without the EP, we reproduce the experimental ITC relative binding free energies excellently (errors of 0.5–1.8 kJ/mol), whereas with the EP parameters, the results are appreciably worse (errors of 3.3–6.2 kJ/mol). This shows that standard AMBER parameters give reliable binding affinities and that it is hard to parametrize an accurate EP model for the halogens.

#### Details of the FEP calculations

In the FEP calculation, we calculated the free energy of alchemically transforming one ligand to another when they are either bound to the protein or are free in solution.^96^ 13 λ values (0.00, 0.05, 0.1, 0.2, …, 0.8, 0.9, 0.95, 1.00) were used in the FEP calculations. At each λ value, the system was subjected to 500 cycles of minimization, followed by 20 ps of equilibration at constant volume and for 1 ns of equilibration at constant pressure. Finally, a 5 ns simulation was run at constant pressure. For the minimization and equilibration at constant volume, all non-hydrogen atoms of the protein, ligand and crystal waters were harmonically restrained toward their starting positions, employing a force constant of 418 kJ/mol/Å^2^. The temperature was kept at 300 K using Langevin dynamics^92^ with a collision frequency of 2 ps^−1^ and the pressure was kept at 1 atm using Berendsen’s barostat.^93^ Periodic boundary conditions were used. A cut-off of 8 Å was used for the non-bonded interactions and the long-range electrostatics were calculated using the particle mesh Ewald (PME) method.^94^

In the FEP calculations, soft-core potentials were used for the van der Waals and electrostatic interactions simultaneously.^97^^,^^98^ A time step of 1 fs was used. Snapshots and energy statistics were collected every 0.5 ps and these were later used to estimate the free energy change with the multistate Bennett acceptance ratio method (MBAR) using the pyMBAR software.^99^ All FEP simulations were run with Amber16.^58^

### Quantification and statistical analysis

#### Isothermal titration calorimetry

A single-site binding model was fitted simultaneously to the 3 titration curves with SEDPHAT^54^ to yield the binding enthalpy (Δ*H*), fraction of binding-competent protein (*n*), and dissociation constant (*K*_d_).

$$\Delta Q_{i}=Q_{i}\;–Q_{i-1}+\left(V_{i}/V_{0}\right)\left[Q_{i}\;–Q_{i-1}\right]/2+Q_{\text{off}}$$

where *V*_*i*_ is the volume of the *i*th injection, *V*_0_ is the cell volume, *Q*_off_ is an offset parameter that accounts for the heat of mixing, and *Q*_i_ is the heat function following the *i*th injection:

$$Q_{i}=\left(\Delta HV_{0}/2\right)\left[\alpha -\sqrt{\alpha^{2}-4nM_{i}X_{i}}\right]$$

where *α* = *nM*_i_ + *X*_i_ + *K*_d_, and *M*_i_ and *X*_i_ are the total concentrations of the protein and the ligand, respectively, in the cell at any given point of the titration. The free energy and entropy of binding were subsequently determined using the relationships Δ*G°* = *RT* ln(*K*_d_) and –*T*Δ*S°* = Δ*G°* – Δ*H°*. SEDPHAT reports asymmetric error estimates, but in the present case we report symmetric estimates, calculated as the average of the upper and lower values, because errors are nearly symmetric for all complexes.

#### FEP calculations

All uncertainties are reported as standard errors of the mean (standard deviations divided by the square root of the number of samples). The pyMBAR software^99^ was used to compute the uncertainty of the MBAR free energies calculated at each *λ* value, using bootstrapping. The simulations were all run in hexaplicate, and the total uncertainty was calculated from the standard error over the six calculations.

It is important to study the convergence of the calculations for all λ values of the FEP calculations.^100^ In order to ensure that the overlap of the studied perturbations was satisfactory, we used the Wu and Kofke bias measure (Π)^101^ to compute the distribution overlap between adjacent λ values. If Π < 0.5, additional intermediate λ values were used.

## Supplemental information

Document S1. Figures S1‒S6 and Tables S1–S3
