# Supplementary material for: Interplay of halogen bonding and solvation in protein–ligand binding
Source: iScience. 2024 Mar 29;27(4):109636. doi: 10.1016/j.isci.2024.109636 (PMC11021960; doi:10.1016/j.isci.2024.109636)

iScience, Volume *27*

Supplemental information

Interplay of halogen bonding

and solvation in protein–ligand binding

Maria Luisa Verteramo, Majda Misini Ignjatović, Rohit Kumar, Sven Wernersson, Vilhelm Ekberg, Johan Wallerstein, Göran Carlström, Veronika Chadimová, Hakon Leffler, Fredrik Zetterberg, Derek T. Logan, Ulf Ryde, Mikael Akke, and Ulf J. Nilsson

Supporting information

Table S1. Collection statistics and model qualities for galectin-3C in complexes S2

Figure S1. ^{1}H-^{15}N HSQC of galectin-3C in complexes and the apo state S3

Figure S2. ^{1}H and ^{15}N chemical shift perturbations versus the enthalpy of binding S4

Figure S3. ^{15}N R_1 relaxation rate constants for the **H**, **F**, **Cl**, **Br**, and **I**-complexes S5

Table S2. Calibration of the EP model for the three ligands with **Cl**, **Br**, and **I** S6

Figure S4. Correlation between the calculated water $-T\Delta S_{tot}$ and the experimental total $-T\Delta S_{ITC}$ S7

Table S3. Comparison of FEP and experimental (ITC) results S8

Figure S5. Superposition of the crystal structures and the water densities from the MD simulations with EP parameters for Cl, Br and I S9

Figure S6. ^{1}H and ^{13}C NMR spectra of **F** S10

Table S1. Collection statistics and model qualities for galectin-3C in complex with **H**, **F**, **Cl**, **Br**, and **I**. Related to figures 3-5.

compound	**H**	**F**	**Cl**	**Br**	**I**
**PDB code**	**6RZI**	**6RZJ**	**6RZK**	**6RZL**	**6RZM**
**station**	BioMAX	BioMAX	BioMAX	BioMAX	BioMAX
**wavelength [Å]**	0.6525	0.6525	0.6525	0.6525	0.6525
**unit cell (Å)**	a = 36.15<br>b = 56.82<br>c = 61.59	a =36.92<br>b = 57.81<br>c = 63.06	a = 36.91<br>b = 57.75<br>c = 63.02	a = 36.57<br>b = 57.63<br>c = 62.85	a = 36.07<br>b= 57.23<br>c= 61.65
**space group**	$P2_12_12_1$	$P2_12_12_1$	$P2_12_12_1$	$P2_12_12_1$	$P2_12_12_1$
**resolution range [Å]**	28.41 - 1.09<br>(1.13 - 1.09)	28.91 - 1.09<br>(1.13 - 1.09)	27.89 - 1.04<br>(1.08 - 1.04)	27.72 - 1.04<br>(1.08 - 1.04)	28.62 - 1.34<br>(1.39 - 1.34)
**completeness [%]**	99.80 (98.37)	99.61 (97.08)	99.82 (98.60)	99.59 (96.35)	99.61 (96.49)
**Total reflections**<br>**unique reflections**	703958 (68572)<br>52849 (5170)	742985 (70431)<br>55916 (5429)	845509 (67945)<br>64248 (6273)	834452 (65960)<br>63350 (6048)	384311 (37592)<br>29030 (2836)
**CC1/2**	1.000 (0.292)	0.999 (0.5)	1.000 (0.692)	0.999 (0.714)	0.999 (0.201)
**multiplicity**	13.3 (13.3)	13.3 (13.0)	13.2 (10.8)	13.2 (10.9)	13.2 (13.3)
**R_{merge} [%]**	0.099 (2.86)	0.104 (1.915)	0.075 (1.219)	0.06253 (1.396)	0.1367 (2.611)
**mean I/σ(I)**	11.62 (0.55)	9.81 (0.66)	14.99 (1.15)	16.18 (1.02)	9.88 (0.51)
**Wilson B-factor [Å²]**	14.04	13.42	11.14	12.34	17.37
**R_{model} (F) [%]**	0.154 (0.318)	0.153 (0.332)	0.128 (0.258)	0.134 (0.299)	0.153(0.344)
**R_{free} (F) [%]**	0.184 (0.354)	0.181 (0.357)	0.152 (0.276)	0.155 (0.293)	0.186(0.384)
**reflections used in refinement (for R_{free})**	52776 (5114)<br>2632 (246)	55788 (5357)<br>2882 (246)	64217 (6262)<br>3242 (301)	63320 (6042)<br>3259 (289)	28937 (2751)<br>1439 (133)
**average B-factors [Å²]**	protein: 16.0<br>ligand: 20.3<br>solvent: 29.3	protein: 15.5<br>ligand: 15.6<br>solvent: 30.1	protein: 12.9<br>ligand: 13.0<br>solvent: 26.7	protein: 15.2<br>ligand: 17.7<br>solvent: 30.5	protein: 18.7<br>ligand: 24.9<br>solvent: 32.4
**Ramachandran outliers [%]**	0.0	0.0	0.0	0.0	0.0
**rotamer outliers [%]**<br>**MolProbity clash score**	0.79<br>1.73	0.0<br>4.59	0.71<br>1.99	0.0<br>3.56	2.16<br>1.22
**bond length rmsd from ideal [Å]**<br>**bond angle rmsd from ideal [°]**	0.013<br>1.27	0.008<br>1.08	0.010<br>1.12	0.009<br>1.08	0.009<br>1.04

Figure S1. ^{1}H-^{15}N HSQC of galectin-3C in complexes. Related to figure 6.

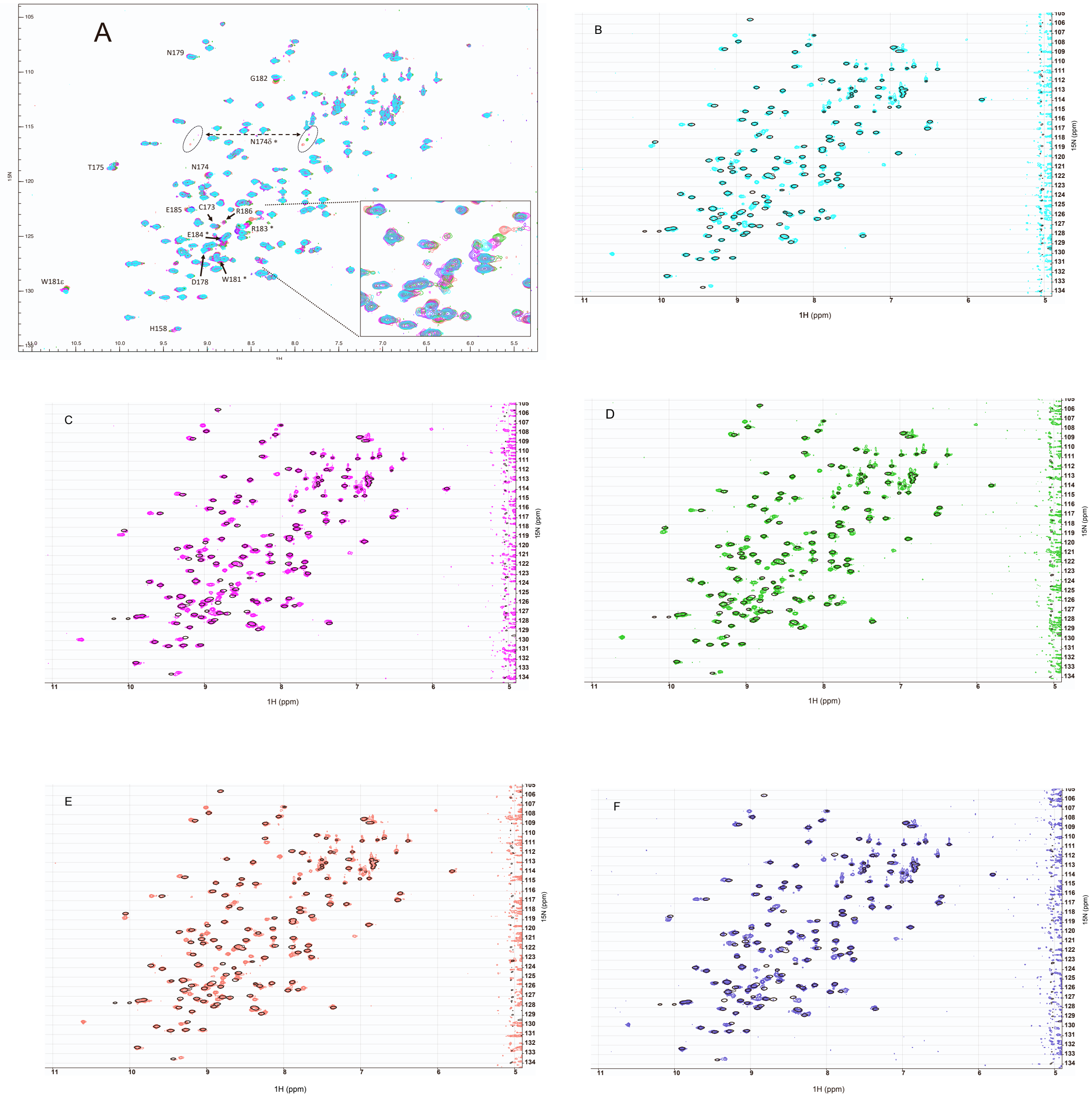

(A) ^{1}H-^{15}N HSQC of galectin-3C in complex with **F** (cyan), **Cl** (magenta), **Br** (green), **I** (pink) and **H** (blue/mauve). Peaks labelled with residue names show significant chemical shift perturbation. Peaks with tentative assignments are marked with a star (*). (B–F) Individual spectra of each complex superimposed on the apo spectrum (black, single contour). (B) **F**, (C) **Cl**, (D) **Br**, (E) **I**, and (F) **H**.

Figure S2. ^{1}H and ^{15}N chemical shift perturbations. Related to figure 6.

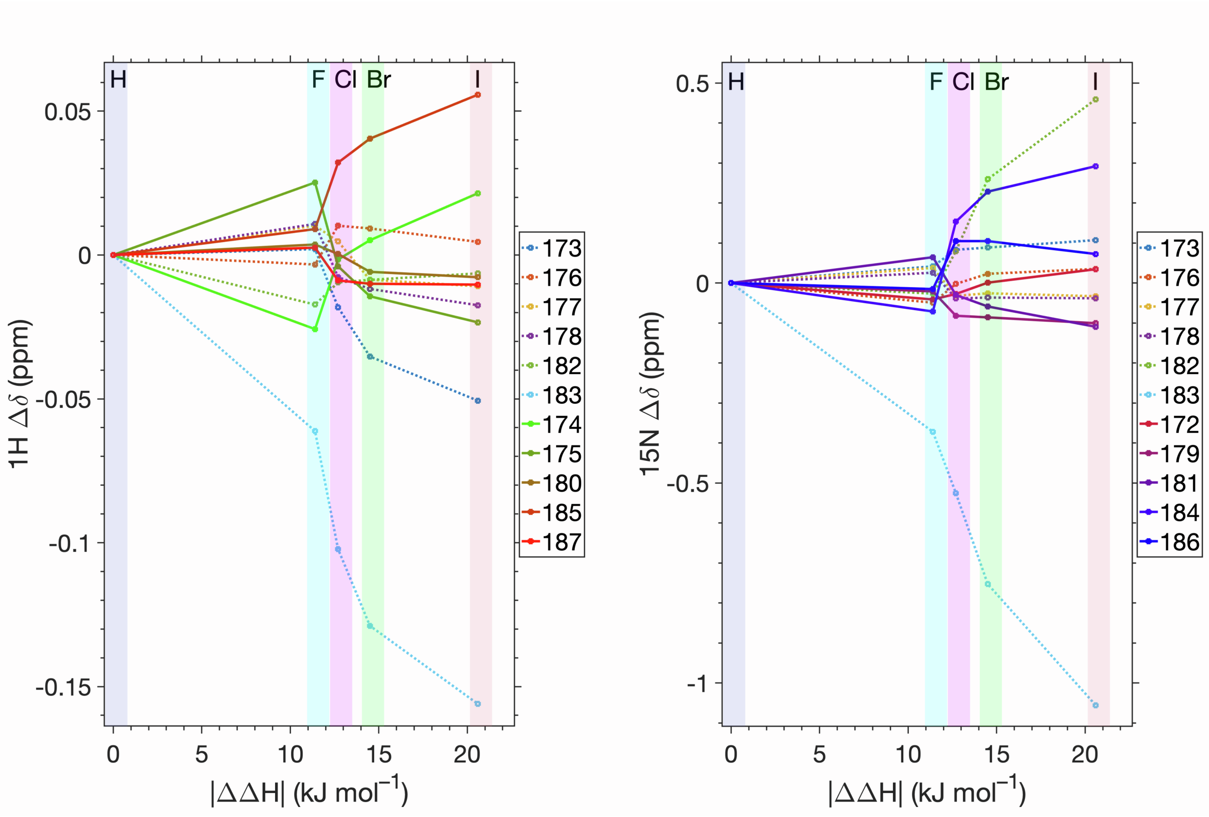

^{1}H and ^{15}N chemical shift perturbations plotted versus the enthalpy of binding $|\Delta\Delta H|$, using data for the **H**–galectin-3C complex as reference. For clarity, only residues with $\Delta\delta \geq 0.01$ are included.

Figure S3. R_1 relaxation rate constants. Related to figure 6.

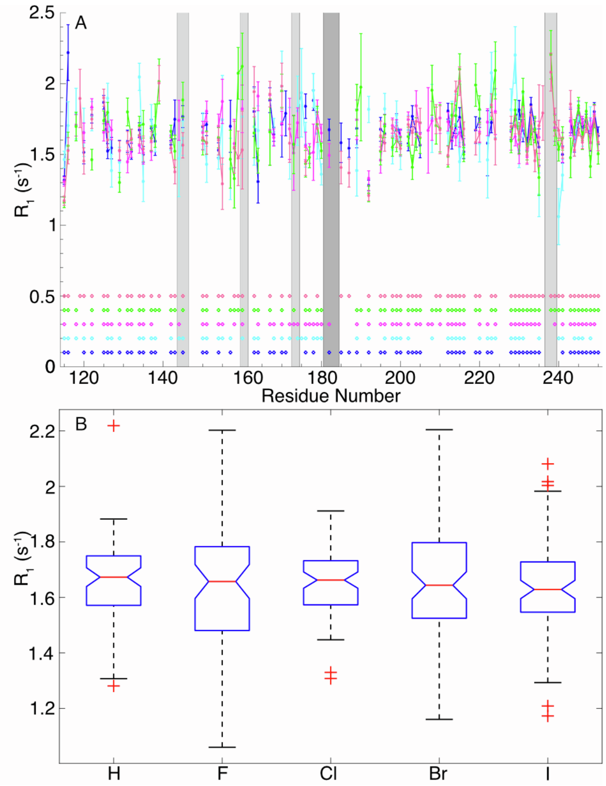

(A) Overview of estimated R_1 relaxation rate constants for all 5 protein-ligand complexes, H (blue), F (teal), Cl (purple), Br (green), and I (pink). The color-coded circles at the bottom of the plot indicate, for each residue, the protein–ligand complexes for which R_1 could be measured. (B) Box plots comparing the distribution of R_1 values for each protein-ligand complex. On each box, the central mark is the median (2nd quantile, q2) and the edges of the box are the 25th and 75th percentiles (1st and 3rd quantiles, q1 and q3, respectively). The whiskers extend to the most extreme data points that are not considered outliers. The outliers are plotted individually using the '+' symbol. The extremes of the whiskers correspond to q3 + 1.5 × (q3 – q1) and q1 – 1.5 × (q3 – q1).

Table S2. Calibration of the EP model for the three ligands **Cl**, **Br**, and **I**. Related to figure 10.

Results of the QM calculation of the ligand–$CH_3NHCOCH_3$ or ligand–H_2O models, as well as the MM calculations with various X–EP distances (Å) are given in terms of the optimum X–OC of X–OH_2 distance (*r* in Å) and interaction energy (Δ*E* in kJ/mol). The best results for the X–OC interactions are marked in bold face, as are the selected X–EP distances.

Cl–EP	Cl–OC		Cl–OH_2		Br–EP	Br–OC		Br–OH_2		I–EP	I–OC		I–OH_2	
	r	Δ*E*	*r*	Δ*E*		*r*	Δ*E*	*r*	Δ*E*		*r*	Δ*E*	*r*	Δ*E*
QM	3.19	-5.7	2.54	-20.8	QM	3.09	-10.2	2.67	-21.2	QM	2.97	-18.7	2.86	-20.4
2.15	**3.17**	-5.2	2.30	-14.1	2.22	2.92	-17.2	2.47	-13.4	2.20	**2.98**	-24.3	2.58	-14.2
2.00	3.13	**-5.8**	2.30	-14.8	2.05	**3.07**	-14.2	2.44	-13.8	**2.15**	3.06	**-20.1**	2.61	-14.2
1.98	3.35	-4.2	2.33	-14.4	**2.02**	**3.11**	-12.7	2.45	-13.7	2.10	3.13	**-17.3**	2.59	-14.2
1.97	3.26	-3.8	2.34	-14.4	2.00	3.11	-12.3	2.42	-13.7	1.95	3.18	-16.4	2.55	-14.8
**1.95**	**3.22**	-4.4	2.33	-14.6	1.82	3.19	-10.5	2.45	-13.9	1.75	3.26	-14.1	2.56	-15.3
1.90	3.30	-3.7	2.31	-14.6	1.78	3.21	**-10.1**	2.43	-14.0	1.55	3.31	-12.6	2.52	-15.7
1.75	3.32	-3.9	2.29	-15.0	1.62	3.26	-9.1	2.50	-14.0					
1.55	3.46	-3.4	2.30	-15.3	1.42	3.31	-8.1	2.41	-14.3					
1.35	3.90	-4.0	2.27	-15.5										

Figure S4. Correlation between the calculated water $-T\Delta S_{tot}$ and the experimental total $-T\Delta S_{ITC}$, using different MM parameters for Cl, Br and I. Related to figure 10.

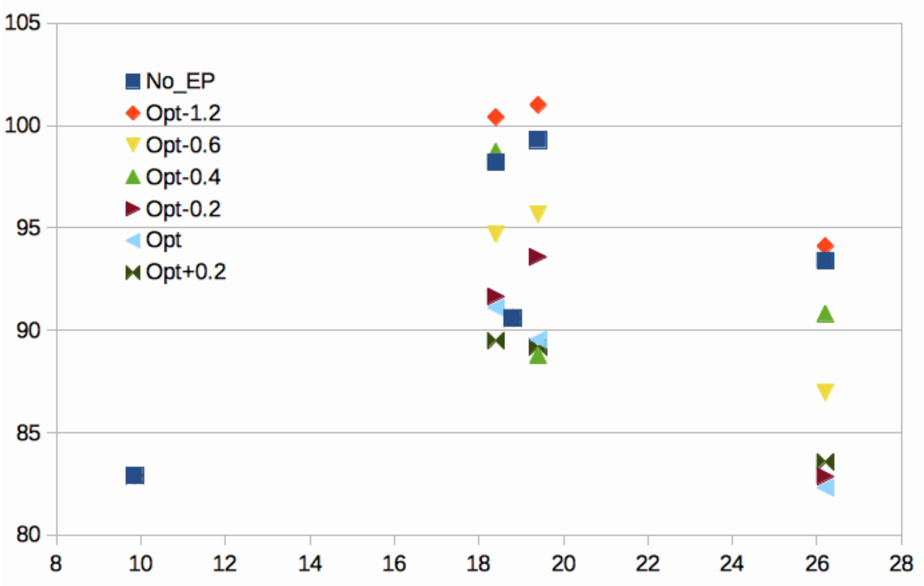

No_EP are the results without any EP charges, presented also in Figure 10 in the main article. The other calculations employ EP with varying values of the X–EP distance around the optimum values (Opt) 1.95, 2.02 and 2.15 Å for X = Cl, Br, and I, respectively.

Table S3 Comparison of FEP and experimental (ITC) results for the relative binding affinities. Related to figure 10.

All energies and uncertainties are given in kJ/mol.

Perturbation	Calculated $\Delta\Delta G$		Experimental
	Without EP	With EP	$\Delta\Delta G$
F → Cl	-1.4 ± 0.8	-7.4 ± 0.8	-1.9 ± 1.1
F → Br	-0.8 ± 0.6	-8.8 ± 1.4	-2.6 ± 1.4
F → I	-3.7 ± 0.9	-5.2 ± 1.0	-1.9 ± 1.1
F → H	1.3 ± 0.4		2.3 ± 0.9

Figure S5. Superposition of the crystal structures and the water densities from the MD simulations. Related to figure 9.

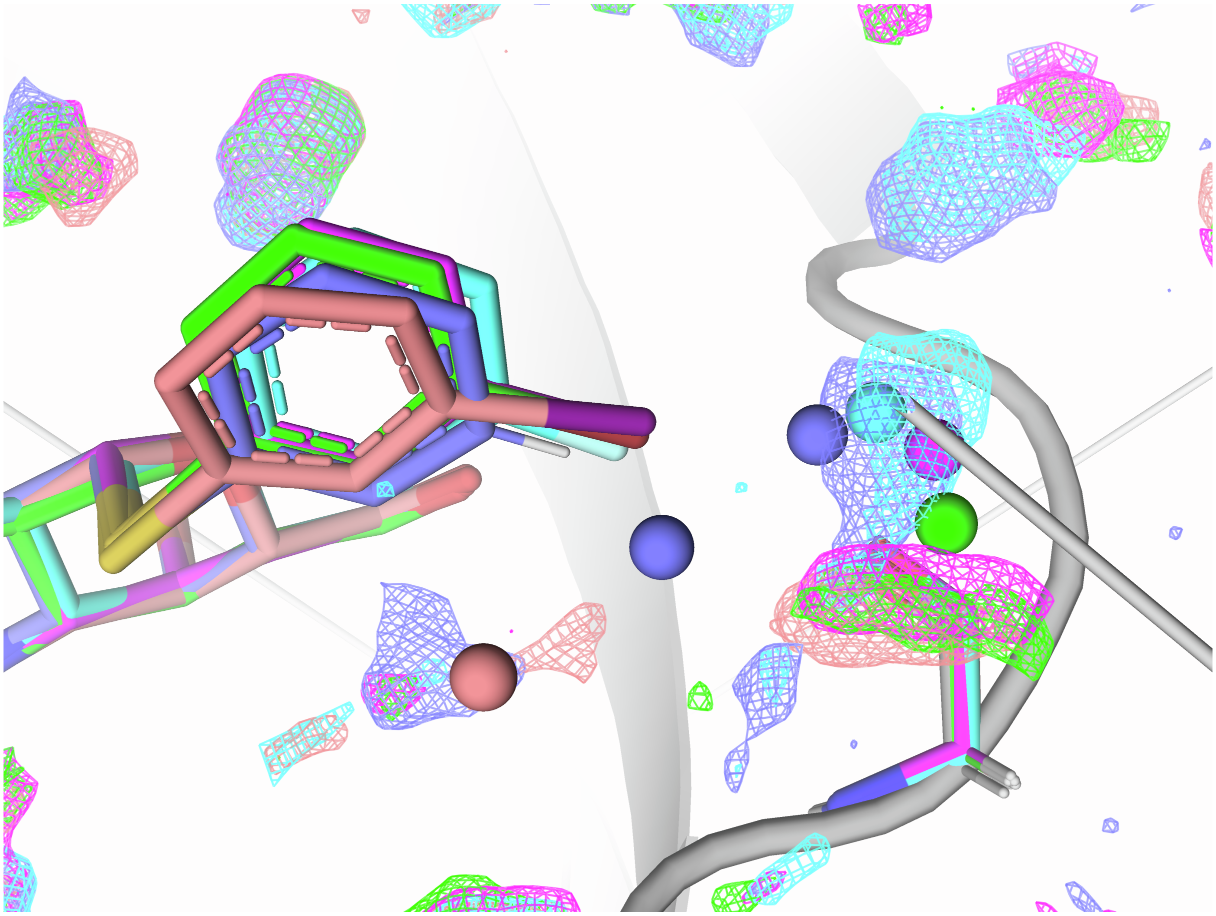

Superposition of the crystal structures and the water densities from the MD simulations for the five galectin-3C–ligand complexes with EP parameters for Cl, Br and I, focused on the variable part of the ligands. The isodensity level is five times the bulk density in all figures. The variable water molecules in the crystal structures are shown as balls. The structures and densities are color coded: **H** (slate), **F** (cyan), **Cl** (magenta), **Br** (green) and **I** (salmon pink).

Figure S6. ^{1}H and ^{13}C NMR spectra of **F.**

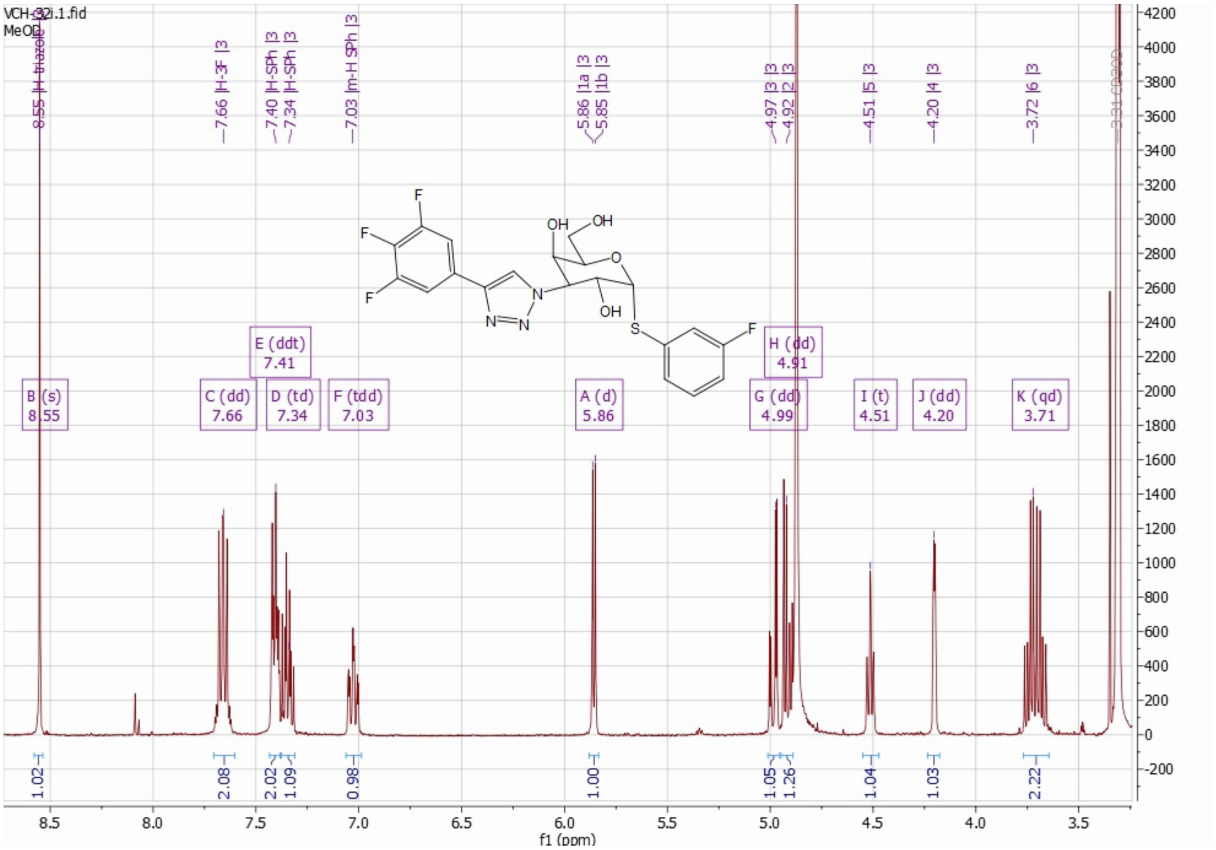

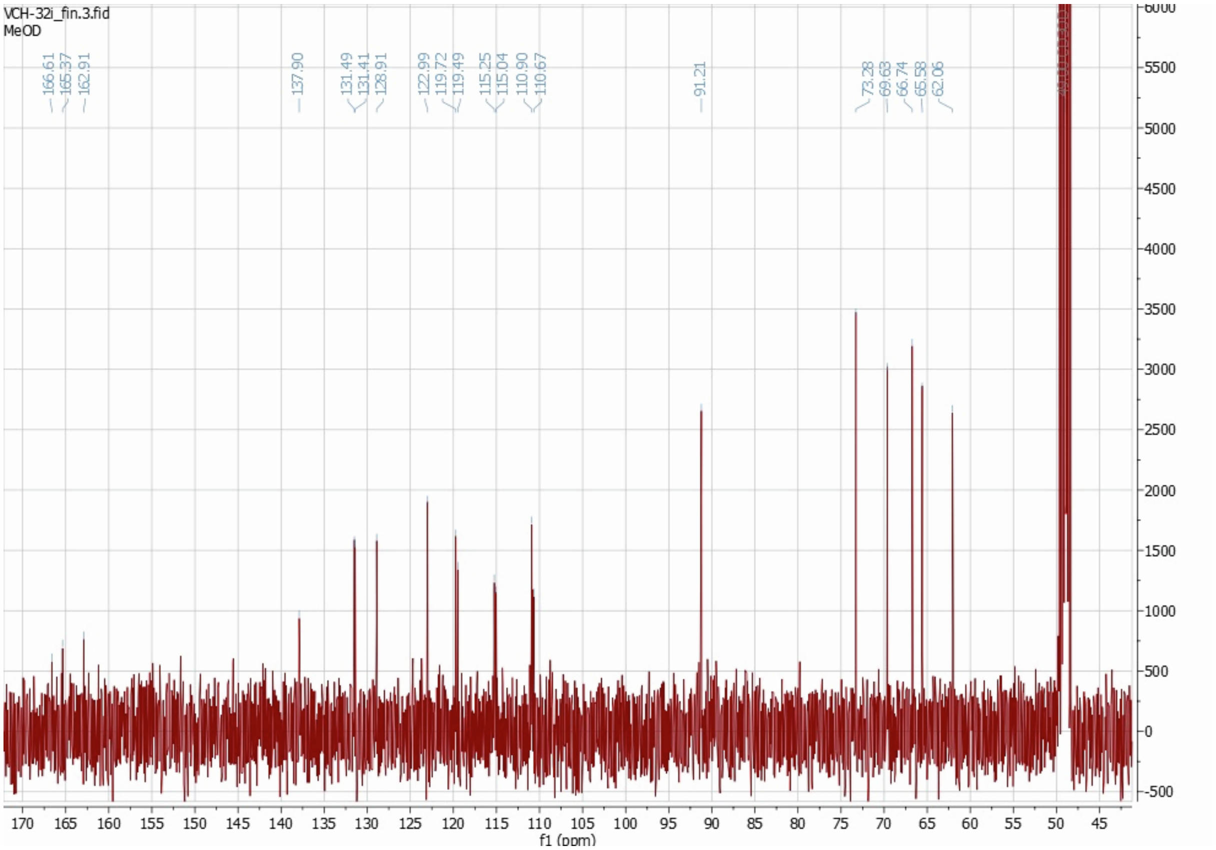

Supplement: Document S1. Figures S1‒S6 and Tables S1–S3 [file mmc1.pdf]
